# Nucleophilic substitution of a phthalimidyl group with morpholine in an *N*^1^-methyl-1,2,3-triazole: crystallographic evidence for migration of the methyl­ene bridge

**DOI:** 10.1107/S2053229626002810

**Published:** 2026-03-24

**Authors:** Paul R. Palme, Richard Goddard, Markus Leutzsch, Adrian Richter, Peter Imming, Rüdiger W. Seidel

**Affiliations:** a Institut für Pharmazie, Martin-Luther-Universität Halle-Wittenberg, Wolfgang-Langenbeck-Strasse 4, 06120 Halle (Saale), Germany; b Max-Planck-Institut für Kohlenforschung, Kaiser-Wilhelm-Platz 1, 45470 Mülheim an der Ruhr, Germany; University of North Texas at Dallas, USA

**Keywords:** 1,2,3-triazole, click chemistry, 1,3-dipolar cyclo­addition, substituent migration, Hirshfeld atom refinement, crystal structure

## Abstract

2-{[4-(4-Bromo­phen­yl)-1*H*-1,2,3-triazol-1-yl]meth­yl}phthalimide, C_17_H_11_BrN_4_O, was synthesized by click chemistry employing a copper-catalyzed azide–alkyne 1,3-dipolar cyclo­addition. The mol­ecule adopts an angular structure and was found to crystallize in the monoclinic system (space group *P*2_1_/*n*, *Z* = 4). Reaction with morpholine in the presence of a base afforded 4-{[4-(4-bromo­phen­yl)-2*H*-1,2,3-triazol-2-yl]meth­yl}morpholine, C_13_H_15_BrN_4_O, as the major product, involving a nucleophilic substitution of morpholine for the phthalimide group and migration of the substituent from N1 to N2 on the 1,2,3-triazole ring. The morpholine-substituted com­pound likewise crystallizes in the monoclinic system (space group *P*2_1_/*c*, *Z* = 4) with the mol­ecule exhibiting an angular shape. Both crystal structures appear to be governed by close packing, with weak C—H⋯O and C—H⋯N hy­dro­gen bonds as the prevailing inter­molecular inter­actions.

## Abstract 2

X-ray crystallography revealed that the reaction of 2-{[4-(4-bromo­phen­yl)-1*H*-1,2,3-triazol-1-yl]meth­yl}phthalimide with morpholine led to substitution of the phthalimide group, including migration of the substituent from N1 to N2 on the 1,2,3-triazole ring.

## Introduction

1,2,3-Triazoles are aromatic heterocyclic com­pounds featuring a five-membered ring with three adjacent N atoms, characterized by their 1*H*- and 2*H*-tautomeric forms (Moura & Tomé, 2022 ▸). The 1,2,3-triazole system has gained much importance in the fields of organic chemistry (Haldón *et al.*, 2015 ▸; Dai *et al.*, 2022 ▸; Vala *et al.*, 2022 ▸), agriculture (Song *et al.*, 2024 ▸), materials science (Verma *et al.*, 2026 ▸) and medicinal chemistry (Bonandi *et al.*, 2017 ▸; Bozorov *et al.*, 2019 ▸; Marzi *et al.*, 2022 ▸; Farwa *et al.*, 2025 ▸). Several 1,2,3-triazole-containing drugs, for example, the cephalosporin anti­biotic cefatrizine, the β-lactamase inhibitor tazobactam, the oxazolidone anti­biotic radezolid, the anti­convulsant rufinamide and the orexin receptor antagonist suvorexant, are on the market.

In medicinal chemistry, inter­est in the 1,2,3-triazole heterocycle can be largely attributed to its capability to serve as a bioisostere for several functional groups (Bonandi *et al.*, 2017 ▸) and as a linking group in 1,2,3-triazole-containing hy­brids. In the latter field, significant progress has been achieved through the development of copper-catalyzed azide–alkyne 1,3-dipolar cyclo­addition (Haldón *et al.*, 2015 ▸), a cornerstone of click chemistry, which enables the facile synthesis of 1,4-regioisomeric 1,2,3-triazoles. A wide variety of synthetic routes to 1,2,3-triazoles and their derivatives, em­ploy­ing various metal catalysts, organocatalysts, as well as catalyst- and solvent-free reactions, have been developed in the past two decades (Vala *et al.*, 2022 ▸).

1,2,3-Triazole-containing hy­brids have attracted our inter­est because of their potential as anti­tubercular agents (Tan *et al.*, 2021 ▸; Scarim & Pavan, 2021 ▸). Herein, we report on the synthesis and structural characterization of an example in which the heterocycle links an *N*-phthalimidylmethyl and a 4-(4-bromo­phen­yl) group. Post-click functionalization of the mol­ecule (Yadav *et al.*, 2025 ▸) with morpholine (Tzara *et al.*, 2020 ▸) resulted in a formal migration of the methyl­ene bridge from N1 to N2 on the triazole ring, while replacing the phthalimide group in a nucleophilic substitution reaction, as proven by X-ray crystallography.

## Experimental

### General

Starting materials and reagents were purchased and used as received. Solvents were distilled before use. The synthesis of *N*-(azido­meth­yl)phthalimide from *N*-(bromo­meth­yl)phthalimide can be found in the literature (Zhang *et al.*, 2013 ▸). The NMR spectrum of **3** in chloro­form-*d* was recorded on a Varian INOVA 500 spectrometer and those of **3** and **4a**/**4b** (see sup­porting information) in aceto­nitrile-*d*_3_ on a Bruker Avance Neo 600 spectrometer (abbreviations: *s* = singlet, *dd* = doublet of doublets and *m* = multiplet). Chemical shifts are reported relative to the residual solvent signals.

### Synthesis and crystallization

#### 2-[4-(4-Bromo­phen­yl)-1*H*-1,2,3-triazol-1-yl)meth­yl]isoindoline-1,3-dione (3)

*N*-(Azido­meth­yl)phthalimide (607 mg, 3.00 mmol) and 1-bromo-4-ethynyl­benzene (543 mg, 3.00 mmol) were suspended in *tert*-butyl alcohol/water (50 ml, 1:1 *v*/*v*) with ultrasonication. CuSO_4_·5H_2_O (7.5 mg, 0.03 mmol) and 0.3 ml of a 1 *M* aqueous solution of sodium ascorbate (0.3 mmol) were added, and the mixture was stirred vigorously overnight until it became clear. Subsequently, water (50 ml) was added and the solution was extracted thrice with ethyl acetate (30 ml). The combined organic layers were dried over anhydrous sodium sulfate. The crude product was purified by flash chromatography (silica gel, di­chloro­methane/methanol gradient) to yield **3** (746 mg, 1.95 mmol, 65%) as a white solid. ^1^H NMR (502 MHz, chloro­form-*d*): δ 8.10 (*s*, 1H), 7.93 (*dd*, *J* = 5.5, 3.1 Hz, 2H), 7.79 (*dd*, *J* = 5.5, 3.1 Hz, 2H), 7.73–7.67 (*m*, 2H), 7.56–7.50 (*m*, 2H), 6.25 (*s*, 2H). ^13^C{^1^H} NMR (126 MHz, chloro­form-*d*): δ 166.7, 147.6, 135.1, 132.1, 131.6, 129.3, 127.5, 124.4, 122.5, 120.7, 50.0. ^1^H NMR (600.20 MHz, aceto­nitrile-*d*_3_): 8.29 (*s*, 1H), 7.91 (*m*, 2H), 7.84 (*m*, 2H), 7.78 (*m*, 2H), 7.59 (*m*, 2H), 6.16 (*s*, 2H). ^13^C{^1^H} NMR (150.94 MHz, aceto­nitrile-*d*_3_): δ 167.0, 147.4, 135.9, 132.9, 132.7, 130.9, 128.4, 124.6, 122.7, 122.4, 51.2. A crystal of **3** suitable for single-crystal X-ray diffraction analysis was obtained from a solution in chloro­form-*d* after the solvent had been evaporated slowly at room temperature.

#### 4-{[4-(4-Bromo­phen­yl)-2*H*-1,2,3-triazol-2-yl]meth­yl}morpholine (4a) and 4-{[4-(4-bromo­phen­yl)-1*H*-1,2,3-tri­az­ol-1-yl)meth­yl]morpholine (4b)

Compound **3** (300 mg, 0.78 mmol) was dissolved in aceto­nitrile (5 ml) and tri­ethyl­amine (506 mg, 5.00 mmol) and mor­pholine (96 mg, 1.10 mmol) were added with stirring. The reaction mixture was heated to 85 °C for 12 h. After cooling to room temperature, water (30 ml) was added. The colourless precipitate so ob­tained was filtered off and dried in the air. A crystal of **4a** suitable for single-crystal X-ray diffraction analysis was ob­tained from a solution in aceto­nitrile after the solvent had evaporated slowly under ambient conditions.

### X-ray crystallography

After initial independent atom model (IAM) refinements with *SHEXL2019* (Sheldrick, 2015*a* ▸), the crystal structures of **3** and **4a** were refined with aspherical atomic form factors using *NoSpherA2* (Kleemiss *et al.*, 2021 ▸; Midgley *et al.*, 2021 ▸) in *OLEX2* (Dolomanov *et al.*, 2009 ▸). Hirshfeld-partitioned electron density was calculated in *ORCA* (Version 5.0; Neese *et al.*, 2020 ▸) using the B3LYP hy­brid functional (Becke, 1993 ▸; Lee *et al.*, 1988 ▸) and the def2-TZVPP basis set (Weigend & Ahlrichs, 2005 ▸). Anisotropic atomic displacement parameters (ADPs) were introduced for all non-H atoms. Positions and isotropic ADPs were refined freely for all H atoms. The ADPs of the Br atom in both **3** and **4a** were refined anharmonically to fourth order using the Gram–Charlier series in *OLEX2*. Although the resolution of the diffraction data does not strictly obey Kuhs’ rule (Kuhs, 1988 ▸), according to which an estimated resolution of (sin θ/λ)_max_ = 1.01 Å^−1^ (*cf* Table 1 ▸) is required to resolve anharmonic atomic displacements, refinement of the Gram–Charlier parameters resulted in flat difference electron-density maps near the Br atoms (Herbst-Irmer *et al.*, 2013 ▸) and in a drop in *wR*(*F*^2^) from 0.0368 to 0.0294 for **3** and from 0.0625 to 0.0571 for **4a**. *F*_o_–*F*_c_(HAR) difference electron-density maps with and without anharmonically refined ADPs for Br atoms, as well as the corresponding Henn–Meindl fractal dimension plots (Meindl & Henn, 2008 ▸), are shown in Fig. S1 in the supporting information. *F*_c_(HAR)–*F*_c_(IAM) deformation density maps and the corresponding *F*_o_–*F*_c_(IAM) difference maps are shown in Fig. S2.

Packing indices were calculated with *PLATON* (Spek, 2020 ▸). Hirshfeld surface analysis was conducted with *CrystalExplorer* (Spackman *et al.*, 2021 ▸), which normalizes *X*—H bond lengths to standard neutron-derived values (Allen & Bruno, 2010 ▸). Crystal data, data collection and structure refinement details are listed in Table 1 ▸.

### Computational methods

Density functional theory (DFT) calculations on the free mol­ecules of **4a** and **4b** were performed using *ORCA* (Version 6.0; Neese, 2025 ▸) with a B3LYP/G (VWN1) hy­brid functional (20% HF exchange) and a def2-TZVPP basis set (Weigend & Ahlrichs, 2005 ▸) with an auxiliary def2/J basis (Weigend, 2006 ▸). The starting geometry for **4a** was taken from the crystal structure, and that for **4b** was built using *Avogadro* (Hanwell *et al.*, 2012 ▸). Optimization of the structures used the BFGS method from an initial Hessian according to Almlöf’s model with a very tight self-consistent field convergence threshold (Fletcher, 2000 ▸). The optimized local minimum-energy structures exhibited only positive modes. A conformational search was performed using the Global Optimization Algorithm (GOAT; de Souza, 2025 ▸) and an extended semi-empirical tight-binding model (Bannwarth *et al.*, 2019 ▸). The six lowest energy conformations so obtained were subsequently optimized as described above and indicate that the conformations described are global minima on the potential energy surface. Cartesian coordinates of the DFT-optimized structures of **4a** and **4b** can be found in the supporting information.

## Results and discussion

### Chemistry

**Figure 1:**
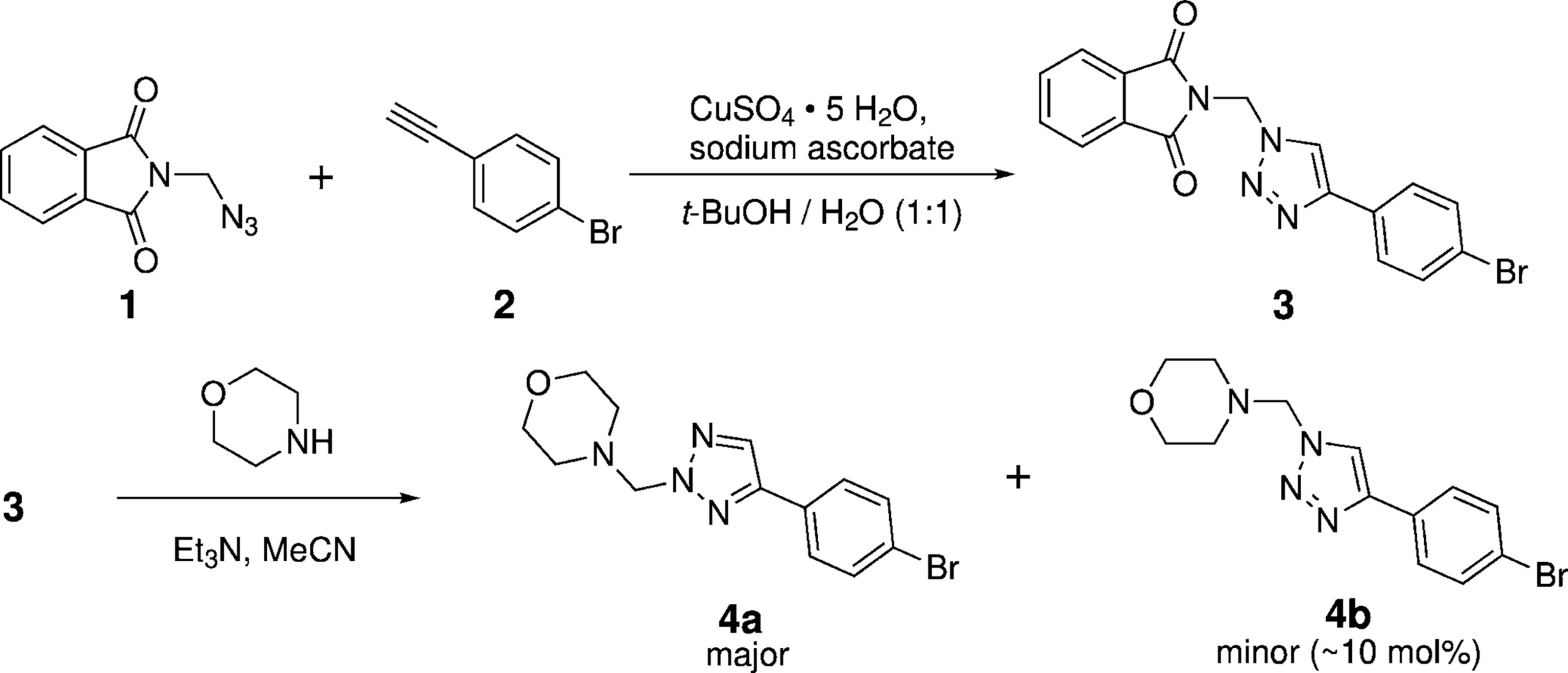
Two-step synthesis of **4a** starting from *N*-(azido­meth­yl)phthalimide (**1**) and 1-bromo-4-ethynyl­benzene (**2**). The amount of the minor com­ponent **4b** was estimated from NMR analysis in aceto­nitrile-*d*_3_ (see supporting information).

The chemistry employed in this work is outlined in Fig. 1 ▸. The 1,2,3-triazole **3** was synthesized from *N*-(azido­meth­yl)phthalimide (**1**) and the terminal alkyne 1-bromo-4-ethynyl­benzene (**2**) using the traditional click-chemistry method, employing a copper-catalyzed 1,3-dipolar cyclo­addition reaction (Haldón *et al.*, 2015 ▸). After flash chromatography, com­pound **3** was obtained in satisfactory yield. ^1^H and ^13^C NMR spectroscopy in aceto­nitrile-*d*_3_ confirmed the structure of the anti­cipated 1,4-regioisomeric 1,2,3-triazole with no signs of isomerization to the *N*^2^-substituted form (Katritzky *et al.*, 2010 ▸). X-ray crystallography established the mol­ecular structure of **3** in the solid state.

In the second step, com­pound **3** was reacted with morpholine in aceto­nitrile in the presence of tri­ethyl­amine as a base. Crystal structure analysis revealed that morpholine replaced the phthalimide group on the methyl­ene group in a nucleophilic substitution reaction, accom­panied by a migration of the methyl­ene bridge from N1 to N2 on the 1,2,3-triazole ring to form the *N*^2^-substituted isomer **4a** as the major com­ponent. Telegina *et al.* (2016 ▸) previously described a similar migration of an *N*^1^-(ferrocenylmeth­yl) group upon alkyl­ation of a 1,2,3-triazole. In the present work, NMR analysis in aceto­nitrile-*d*_3_ showed that the *N*^1^-substituted isomer **4b** was present as a minor com­ponent (see supporting information), which could not be separated and crystallographically characterized in the present work. It is known that *N*-(α-amino­alk­yl)-1,2,3-triazoles can isomerize between their *N*^1^- and *N*^2^-substituted isomers in solution. The equilibrium depends on the electron-withdrawing effect of the groups bonded to the amino N atom and the polarity of the solvent (Katritzky *et al.*, 2010 ▸).

According to DFT calculations on the free mol­ecules, isomer **4a** is more stable than **4b** by 5 kcal mol^−1^. This energy difference is similar to that reported for *N*^1^- and *N*^2^-(ferrocenylmeth­yl)-substituted 1,2,3-triazoles (Telegina *et al.*, 2016 ▸). For the parent 1,2,3-triazole, the 2*H*-tautomer was reported to be about 4.5 kcal mol^−1^ more stable than the 1*H*-tautomer in the gas phase (Katritzky *et al.*, 2010 ▸).

### Crystal and mol­ecular structure of 3

**Figure 2:**
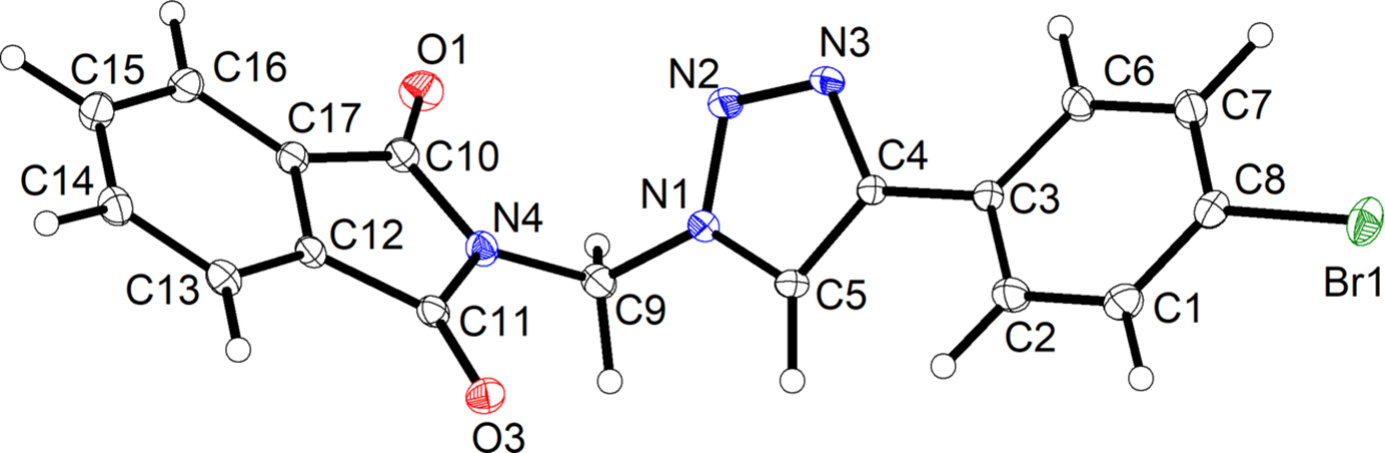
The mol­ecular structure of **3** in the crystal. Displacement ellipsoids are drawn at the 50% probability level. H atoms are shown as small spheres of arbitrary radius.

Fig. 2 ▸ depicts the mol­ecular structure of **3** in the crystal. The triazole ring and the benzene ring are significantly twisted about the C3—C4 bond, with an angle between the respective mean planes of 23.12 (4)°. The phthalimide moiety is virtually planar and the angle between its mean plane and that of the triazole ring linked by the methyl­ene bridge is 69.45 (3)°, resulting in an angular structure of the mol­ecule, as also encountered in the crystal structure of the related *N*^1^-(1*H*-1,2,3-benzotriazol-1-ylmeth­yl)phthalimide (CSD refcode HOFPEY; Wang *et al.*, 2008 ▸).

**Figure 3:**
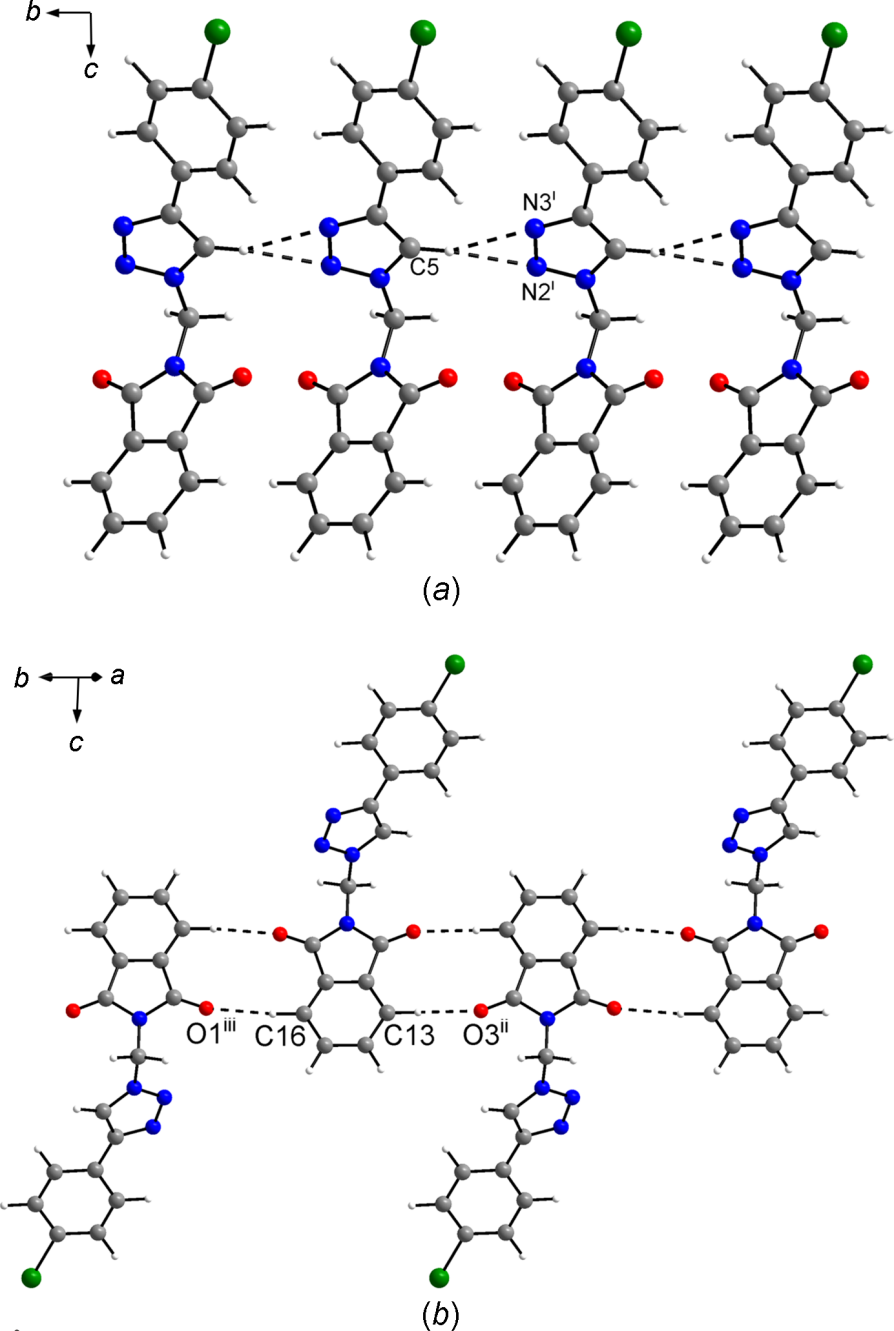
Sections of the crystal structure of **3**, viewed (*a*) along the *a*-axis direction and (*b*) along the [110] direction. Dashed lines represent weak hy­dro­gen bonds. Colour scheme: C grey, H white, Br green, N blue and O red. [Symmetry codes: (i) *x*, *y* − 1, *z*; (ii) −*x* + 2, −*y* + 1, −*z* + 2; (iii) −*x* + 1, −*y* + 3, −*z* + 2.]

In the crystal, the mol­ecules are densely packed with a Kitajgorodskij packing index (Kitajgorodskij, 1973 ▸) of 73.1%. Although close packing seems to dominate the solid-state structure, some inter­molecular contacts shorter than the sum of the corresponding van der Waals radii (Bondi, 1964 ▸) are indicative of weak hy­dro­gen bonds (Table 2 ▸). The triazole C—H group forms a donating weak bifurcated C—H⋯N hy­dro­gen bond to the N atoms of the triazole moiety of a neighbouring mol­ecule related by translational symmetry in the crystallographic *b*-axis direction. The phthalimide groups of adjacent mol­ecules are each joined *via* two weak C—H⋯O hy­dro­gen bonds between the C—H groups in the 4- and 7-positions, and the carbonyl O atoms with a centrosymmetric 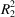
(10) motif (Bernstein *et al.*, 1995 ▸), resulting in tapes along the [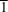
20] direction (Fig. 3 ▸). Halogen bonding is absent in the crystal structure of **3**.

### Crystal and mol­ecular structure of 4a

**Figure 4:**
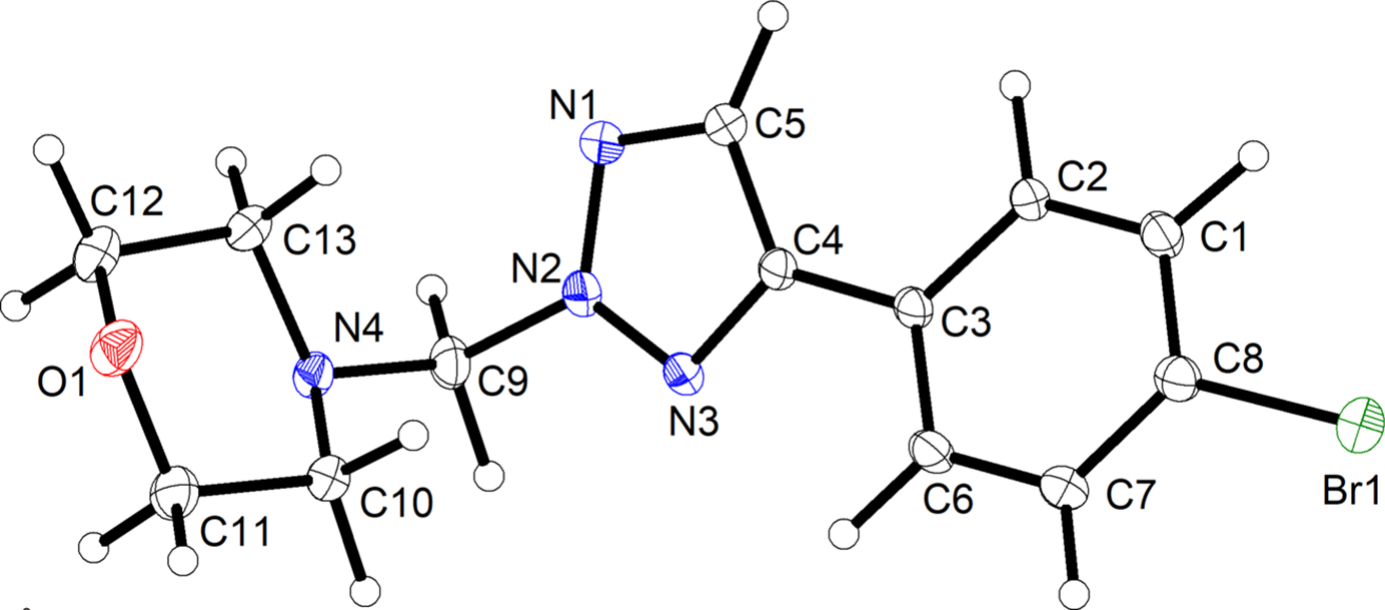
The mol­ecular structure of **4a** in the crystal. Displacement ellipsoids are drawn at the 50% probability level. H atoms are shown as small spheres of arbitrary radius.

Fig. 4 ▸ shows the mol­ecular structure of **4a** in the crystal. The tilt angle between the mean planes through the triazole ring and the benzene ring is slightly less than in **3** at 18.3 (1)°. As expected, the morpholine six-membered aliphatic heterocycle adopts a low-energy chair conformation, with the *N*-methyl­ene group in the equatorial position, resulting in an angular shape of the mol­ecule.

**Figure 5:**
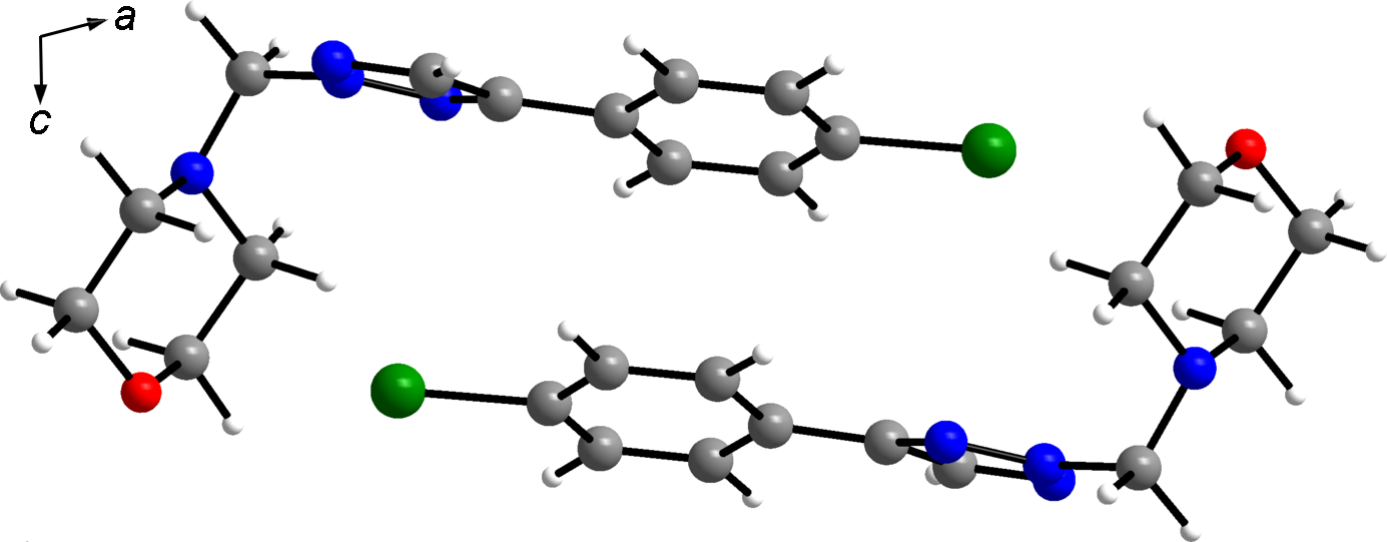
Centrosymmetric dimeric arrangement of the mol­ecules in the crystal structure of **4a**, viewed along the *b*-axis direction. Colour scheme: C grey, H white, Br green, N blue and O red.

**Figure 6:**
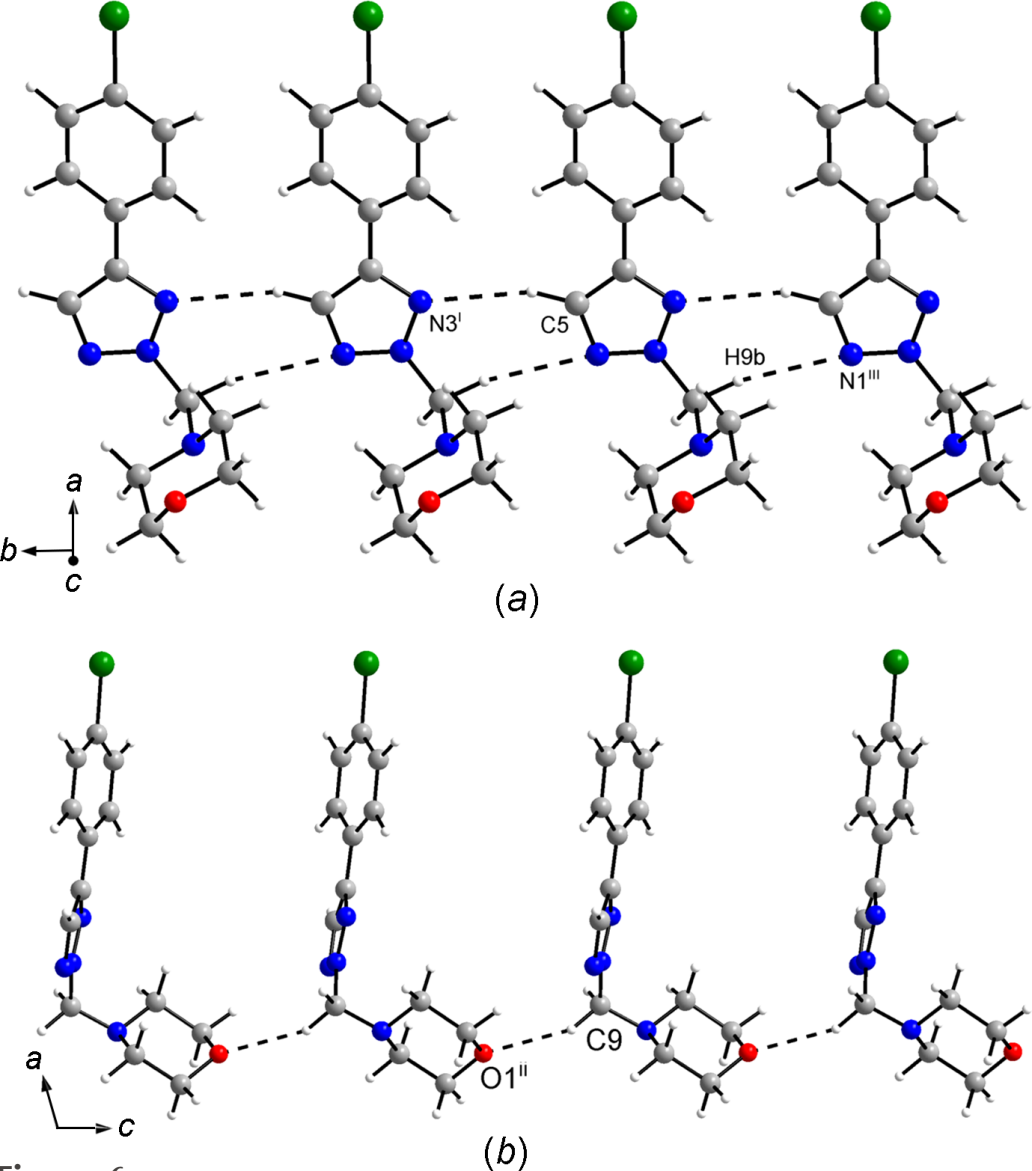
Sections of the crystal structure of **4a**, viewed (*a*) approximately along the *c*-axis direction and (*b*) along the *b*-axis direction. Dashed lines represent weak hy­dro­gen bonds. Colour scheme: C grey, H white, Br green, N blue and O red. [Symmetry codes: (i) *x*, *y* + 1, *z*; (ii) *x*, −*y* + 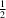, *z* − 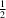; (iii) *x* + 1, *y* − 1, *z*.]

A Kitajgorodskij packing index (Kitajgorodskij, 1973 ▸) of 73.1% was calculated for **4a**, likewise indicating a dense crystal packing. The central motif in the crystal packing is a centrosymmetric dimeric arrangement of the mol­ecules (Fig. 5 ▸). The separation between the mean planes through the benzene rings in the mol­ecules constituting a dimer is 3.55 Å and the corresponding centroid–centroid distance is 3.724 (1) Å, which is typical of face-to-face aromatic stacking. Within a dimer, the morpholine O atom approaches the Br atom of the symmetry-related mol­ecule, but the O⋯Br distance (longer than the sum of the corresponding van der Waals radii at 3.71 Å; Bondi, 1964 ▸) and the O⋯Br—C angle (150.2°) are not indicative of halogen bonding. The crystal structure features several C—H⋯N and C—H⋯O short contacts that can be regarded as weak inter­molecular hy­dro­gen bonds (Table 3 ▸). Worth noting are those formed between the triazole C—H group and an N atom of the triazole ring of an adjacent mol­ecule (C5—H5⋯N3^i^) and those formed between the two methyl­ene C—H groups and the morpholine O atom (C9—H9*A*⋯O1^ii^) and a triazole N atom (C9—H9*B*⋯N1^iii^) of neighbouring mol­ecules (Fig. 6 ▸).

A search of the Cambridge Structural Database (CSD; Groom *et al.*, 2016 ▸) revealed only a few examples of structurally characterized 2-amino­methyl-1,2,3-triazoles related to **4a**. For example, 1-(4-bromo­phen­yl)-*N*-methyl-*N*-[(4-phenyl-2*H*-1,2,3-triazol-2-yl)meth­yl]methanamine (CSD refcode ZET­XII; Gupta *et al.*, 2018 ▸) and *N*,*N*-dibenzyl-1-[4-(4-fluoro­phen­yl)-2*H*-1,2,3-triazol-2-yl]methanamine (AKITEV; Jiang *et al.*, 2016 ▸). Like in **4a**, the N—N—C—N torsion angle in these structures is close to 90 °C.

### Hirshfeld surface analysis

**Figure 7:**
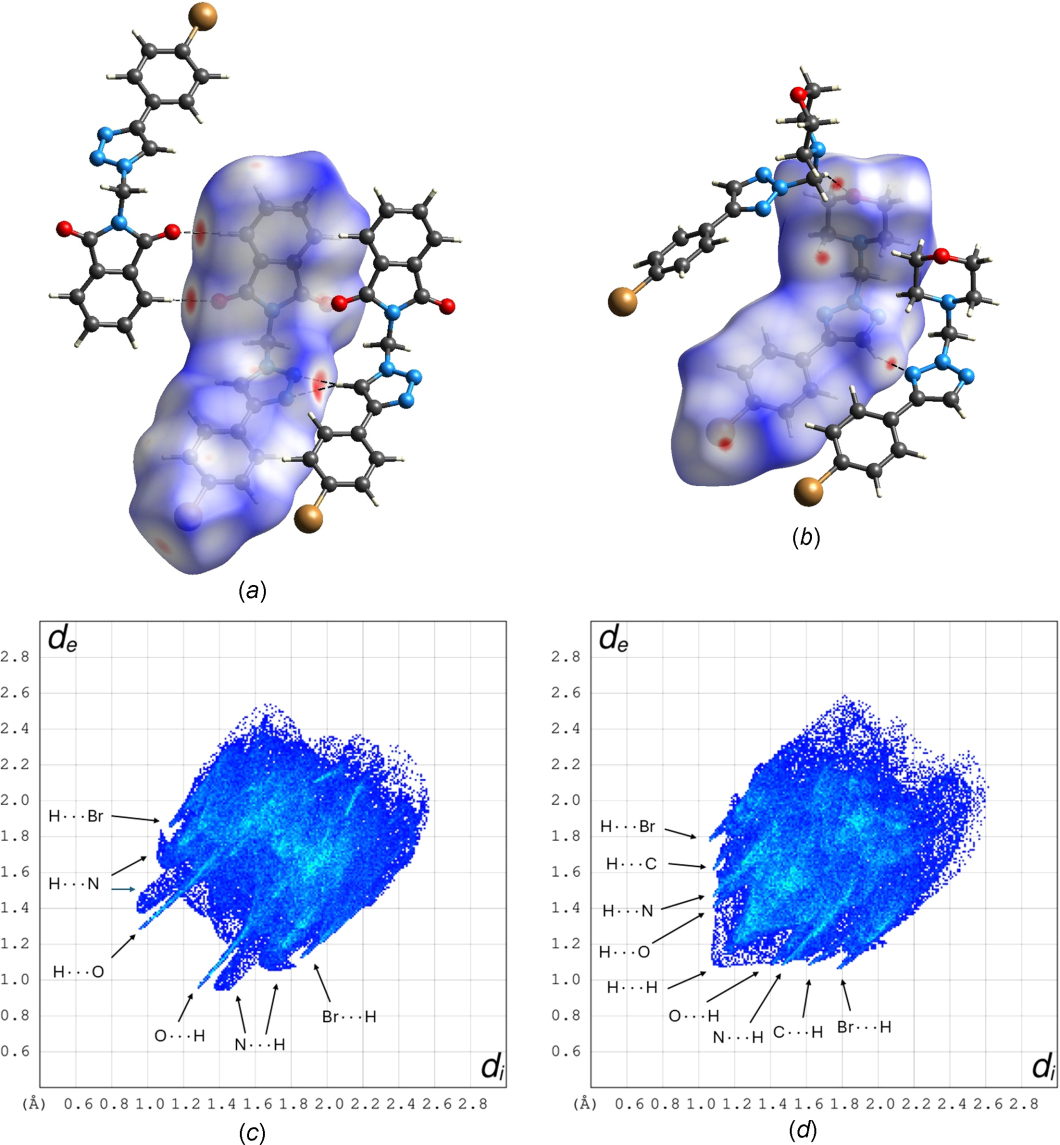
Hirshfeld surface mapped with *d*_norm_ for (*a*) **3** and (*b*) **4a**, and the corresponding fingerprint plots (*c*) and (*d*). *d*_i_ is the distance from a point on the Hirshfeld surface to the nearest nucleus inside the surface (*i.e.* belonging to the reference mol­ecule) and *d*_e_ is the distance from the same surface point to the nearest nucleus outside the surface (*i.e.* in any neighbouring mol­ecule). Dashed lines show weak hy­dro­gen bonds. Colour scheme for the atoms: C grey, H white, Br bronze, N blue and O red.

In order to gain insight into the mol­ecular environments of **3** and **4a** in the crystal structures, we performed Hirshfeld surface analyses (Spackman & Jayatilaka, 2009 ▸). Figs. 7 ▸(*a*) and 7(*b*) show the Hirshfeld surfaces for **3** and **4a** mapped with the normalized contact distance (*d*_norm_), whereby the colours indicate inter­molecular contacts shorter (red), approximately equal (white) or longer (blue) than the sum of the van der Waals radii. The pronounced red-coloured concave areas on the Hirshfeld surface for **3** [Fig. 7 ▸(*a*)] correspond to the weak C—H⋯O and bifurcated C—H⋯N hy­dro­gen bonds described in Section 3.2. Inspection of the *d*_norm_ plot for **4a** reveals that the C—H⋯O and C—H⋯N inter­actions are less significant than in **3**; the respective red areas on the Hirshfeld surface are smaller [Fig. 7 ▸(*b*)].

As expected, the corresponding 2D fingerprint plot for **3** shows characteristic spikes of the O⋯H/H⋯O contacts resulting from the weak C—H⋯O hy­dro­gen bonds. The N⋯H/H⋯N contacts from the weak bifurcated C—H⋯N hy­dro­gen bonds give rise to more diffuse wing-like features. In contrast, the fingerprint plot for **4a** is more diffuse as a whole than that for **3**, indicating that H⋯H contacts from close packing are more frequent (44.6% of the surface area) than in **3** (21.9%). Whereas small spikes from Br⋯H/H⋯Br contacts are present for both structures, C⋯H/H⋯C contacts resulting from edge-to-face (C—H⋯π) aromatic stacking are observed only for **4a**. A triangular feature on the centre of the diagonal characteristic of face-to-face aromatic stacking is not pronounced either in **3** and **4a**.

## Conclusions

In this article, we have reported the synthesis of the 1,2,3-triazole **3** bearing an *N*^1^-phthalimidylmethyl group through click chemistry and its structural characterization by X-ray crystallography. The crystal structure of **3** features weak inter­molecular hy­dro­gen bonds of the C—H⋯O type between the phthalimidyl groups and of the C—H⋯N type between the triazole rings of adjacent mol­ecules. X-ray crystallography provided clear evidence that the bridging methyl­ene group underwent a migration from N1 to N2 on the triazole ring in the major product **4a**, resulting from a nucleophilic substitution of the phthalimidyl group in **3** with morpholine. DFT calculations on the free mol­ecules indicate that **4a** is lower in energy than the *N*^1^-substituted structural isomer **4b**, which was detected as a minor product. Examples of similar migrations of nitro­gen-bound substituents on 1,2,3-triazoles appear to be scarce.

## Supplementary Material

Crystal structure: contains datablock(s) 3, 4a, global. DOI: 10.1107/S2053229626002810/yd3069sup1.cif

Structure factors: contains datablock(s) 3. DOI: 10.1107/S2053229626002810/yd30693sup2.hkl

Supporting information file. DOI: 10.1107/S2053229626002810/yd30693sup4.cdx

Structure factors: contains datablock(s) 4a. DOI: 10.1107/S2053229626002810/yd30694asup3.hkl

Supporting information file. DOI: 10.1107/S2053229626002810/yd30694asup5.cdx

Supporting information file. DOI: 10.1107/S2053229626002810/yd30693sup6.cml

Supporting information file. DOI: 10.1107/S2053229626002810/yd30694asup7.cml

Difference electron-density maps. DOI: 10.1107/S2053229626002810/yd3069sup8.pdf

NMR spectra. DOI: 10.1107/S2053229626002810/yd3069sup9.pdf

Cartesian coordinates of the DFT-optimized structure of 4a. DOI: 10.1107/S2053229626002810/yd3069sup10.txt

Cartesian coordinates of the DFT-optimized structure of 4b. DOI: 10.1107/S2053229626002810/yd3069sup11.txt

CCDC references: 2538228, 2538227

## Figures and Tables

**Table 1:** Experimental details For both structures: *Z* = 4. Experiments were carried out at 100 K with Mo *K*α radiation. The absorption correction was Gaussian (*SADABS*; Bruker, 2016 ▸). All H-atom parameters were refined.

	**3**	**4a**
Crystal data		
Chemical formula	C_17_H_11_BrN_4_O_2_	C_13_H_15_BrN_4_O
*M*_r_	383.21	323.19
Crystal system, space group	Monoclinic, *P*2_1_/*n*	Monoclinic, *P*2_1_/*c*
*a*, *b*, *c* (Å)	8.9293 (5), 5.4537 (3), 31.0109 (16)	16.5886 (11), 5.7516 (4), 14.3116 (11)
β (°)	93.347 (2)	104.348 (2)
*V* (Å^3^)	1507.58 (14)	1322.89 (16)
μ (mm^−1^)	2.75	3.11
Crystal size (mm)	0.52 × 0.07 × 0.04	0.08 × 0.02 × 0.02

Data collection		
Diffractometer	Bruker Kappa Mach3 APEXII	Bruker D8 Venture
*T*_min_, *T*_max_	0.607, 0.906	0.872, 0.960
No. of measured, independent and observed [*I* ≥ 2σ(*I*)] reflections	56275, 5290, 4650	197950, 3296, 2614
*R*_int_	0.037	0.158
(sin θ/λ)_max_ (Å^−1^)	0.750	0.668

Refinement		
*R*[*F*^2^ > 2σ(*F*^2^)], *wR*(*F*^2^), *S*	0.018, 0.029, 1.06	0.026, 0.057, 1.07
No. of reflections	5290	3296
No. of parameters	286	257
Δρ_max_, Δρ_min_ (e Å^−3^)	0.31, −0.29	0.58, −0.49

Computer programs: *APEX5* (Bruker, 2022 ▸), *SAINT* (Bruker, 2019 ▸), *SHELXT* (Sheldrick, 2015*b* ▸), *olex2.refine* (Bourhis *et al.*, 2015 ▸), *DIAMOND* (Brandenburg, 2018 ▸) and *publCIF* (Westrip, 2010 ▸).

**Table 2:** Hydrogen-bond geometry (Å, °) for **3**

*D*—H⋯*A*	*D*—H	H⋯*A*	*D*⋯*A*	*D*—H⋯*A*
C5—H5⋯N2^i^	1.051 (9)	2.431 (9)	3.4527 (9)	163.8 (7)
C5—H5⋯N3^i^	1.051 (9)	2.356 (9)	3.3625 (9)	160.0 (7)
C13—H13⋯O3^ii^	1.058 (9)	2.259 (9)	3.2941 (9)	165.5 (7)
C16—H16⋯O1^iii^	1.075 (9)	2.370 (9)	3.4281 (9)	167.9 (7)

Symmetry codes: (i) 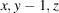; (ii) 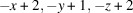; (iii) 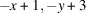, 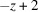.

**Table 3:** Hydrogen-bond geometry (Å, °) for **4a**

*D*—H⋯*A*	*D*—H	H⋯*A*	*D*⋯*A*	*D*—H⋯*A*
C5—H5⋯N3^i^	1.06 (3)	2.57 (3)	3.572 (3)	158.3 (19)
C9—H9*A*⋯O1^ii^	1.11 (2)	2.47 (2)	3.481 (2)	150.3 (15)
C9—H9*B*⋯N1^iii^	1.10 (2)	2.66 (2)	3.696 (3)	157.7 (17)
C11—H11*A*⋯O1^iv^	1.08 (2)	2.67 (2)	3.426 (3)	126.2 (16)
C12—H12*A*⋯O1^v^	1.07 (2)	2.62 (2)	3.661 (2)	166.0 (16)
C13—H13*A*⋯N4^vi^	1.08 (2)	2.63 (2)	3.553 (2)	142.4 (16)

Symmetry codes: (i) 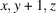; (ii) 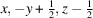; (iii) 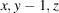; (iv) 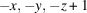; (v) 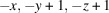; (vi) 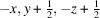.
