# Supplementary material for: Nucleophilic substitution of a phthalimidyl group with morpholine in an N1-methyl-1,2,3-triazole: crystallographic evidence for migration of the methyl­ene bridge
Source: Acta Crystallogr C Struct Chem. 2026 Mar 24;82(Pt 4):144–50. doi: 10.1107/S2053229626002810 (PMC13051533; doi:10.1107/S2053229626002810)
Supplement: Supplementary file 8 [file c-82-00144-sup8.pdf]

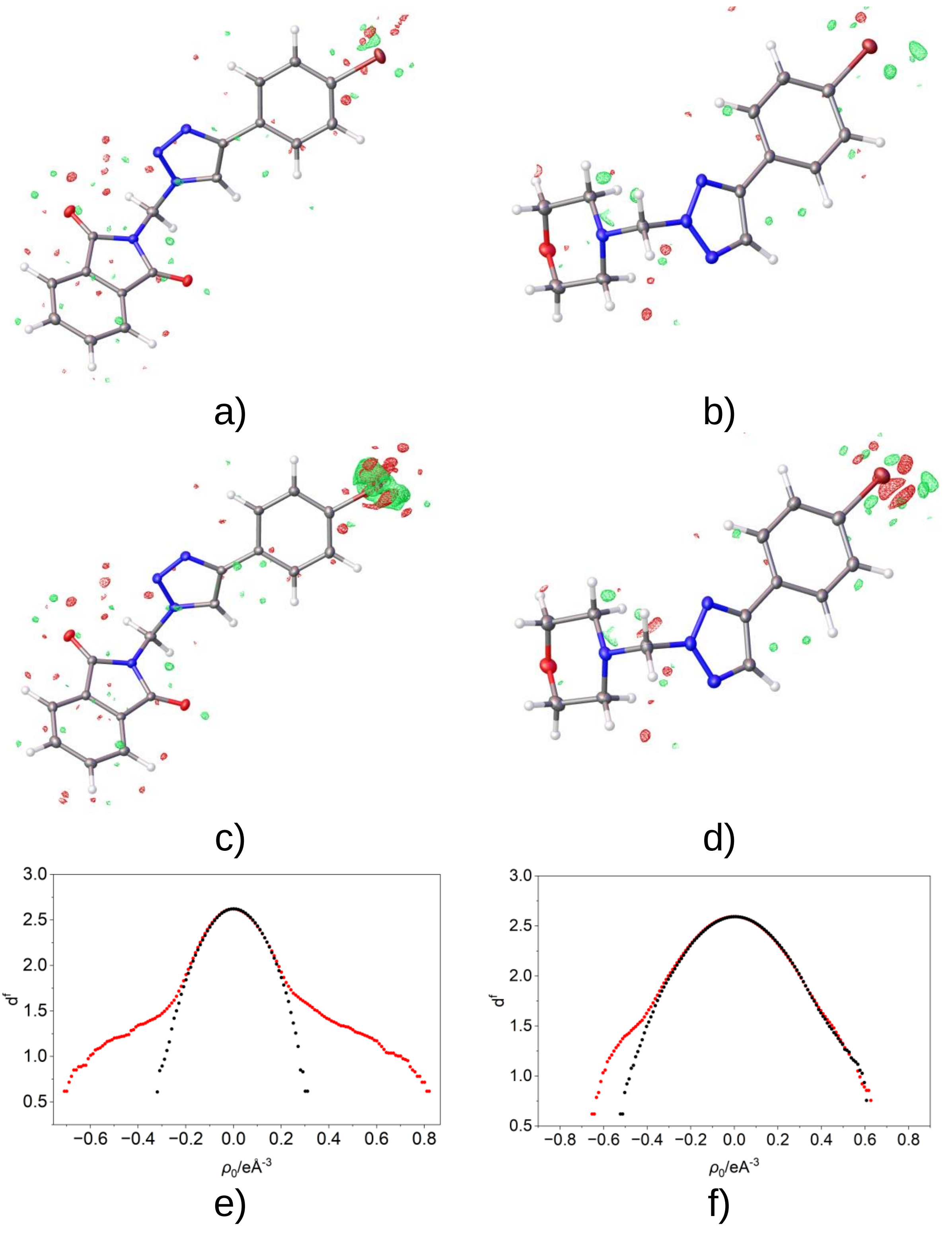


**Figure S1**. $F_o$–$F_c$(HAR) difference electron density with a fourth order Gram-Charlier correction for anharmonic displacement of Br1 a) for **3** and b) for **4a** and c), d) without the correction (plotted on a grid of 0.1 Å with iso-values of 0.20 eÅ$^{-3}$ for **3** and 0.40 eÅ$^{-3}$ for **4a**; green = positive, red = negative). Parts e) and f) show the corresponding Henn-Meindl fractal dimension plots (red without correction, black with correction).

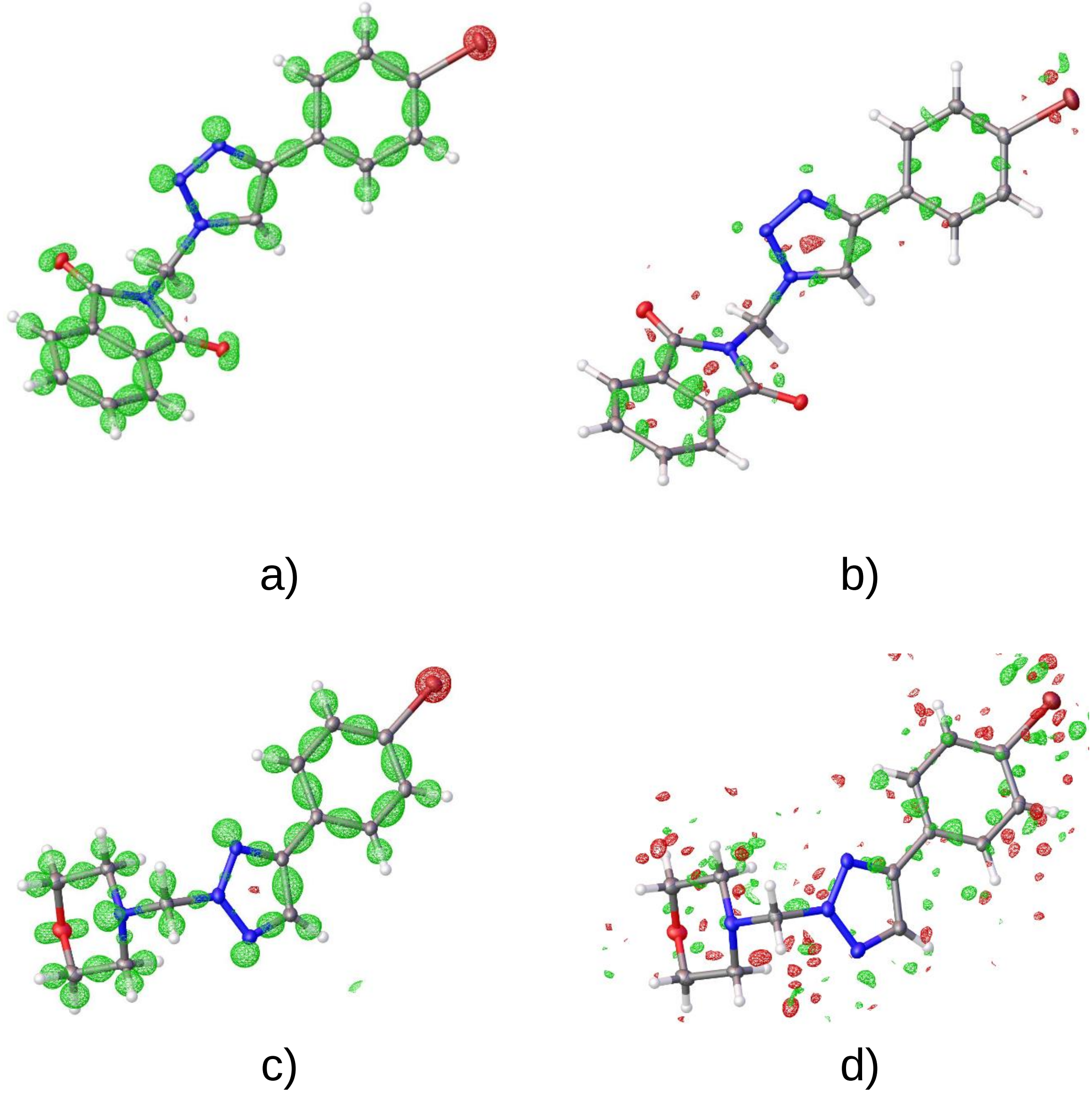


**Figure S2**. a) $F_c$(HAR)–$F_c$(IAM) deformation density map for **3** (map level 0.23 eÅ$^{-3}$; green = positive, red = negative). and b) the corresponding $F_o$–$F_c$(IAM) (map level 0.23 eÅ$^{-3}$). c) $F_c$(HAR)–$F_c$(IAM) difference electron density map for **4a** (map level 0.21 eÅ$^{-3}$) and d) the corresponding $F_o$–$F_c$(IAM) (map level 0.32 eÅ$^{-3}$).

Application of a $4^{th}$ order Gram-Charlier anharmonic model for the Br atom in both structures resulted in negative PDFs (**3**: negative probability of 0.54% to find the Br atom in a 2815 $pm^3$ volume at a distance of 0.445 Å from the atom; **4a**: negative probability of 4.01% to find the Br atom in a 7191 $pm^3$ volume at a distance of 0.433 Å from the atom), but a decrease in the maximum to minimum residual electron density range (from max/min 0.7276/–0.5275 to 0.3072/–0.2829 in **3** and from max/min 0.5705/–0.5961 to 0.5810/–0.4858 in **4a**), and the standard uncertainties in some of the bond distances and angles were improved, in particular in those of the C–H bond lengths. In both cases, the weighted *R* values decreased (from 0.037 to 0.030 for all reflections in **3** and from 0.062 to 0.056 for all reflections in **4a**).
