# Supplementary material for: Nucleophilic substitution of a phthalimidyl group with morpholine in an N1-methyl-1,2,3-triazole: crystallographic evidence for migration of the methyl­ene bridge
Source: Acta Crystallogr C Struct Chem. 2026 Mar 24;82(Pt 4):144–50. doi: 10.1107/S2053229626002810 (PMC13051533; doi:10.1107/S2053229626002810)

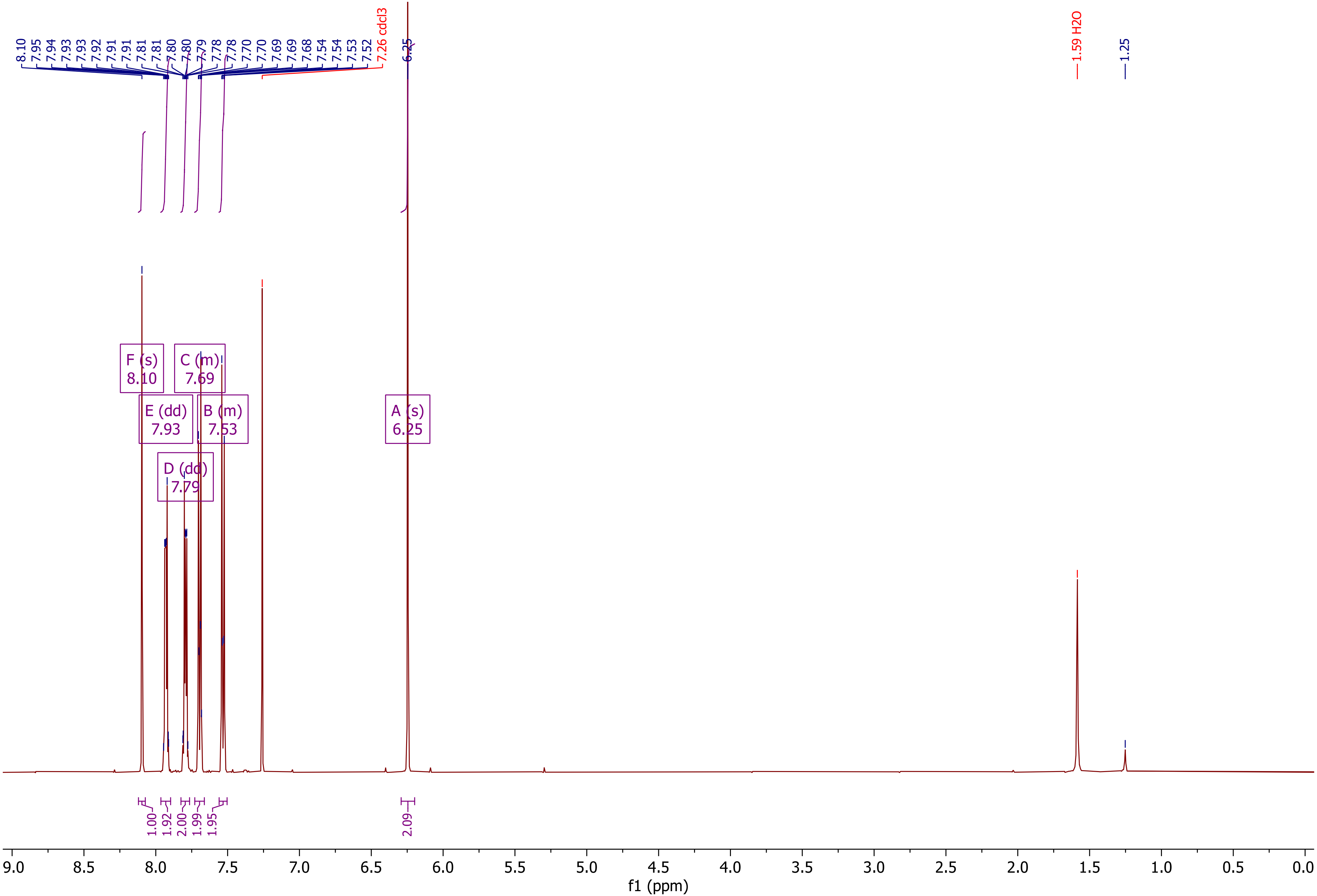

Palme_6064-67_PROTON_2023-11-03_02 — 2023-11-02 — Palme_6064-67 — —

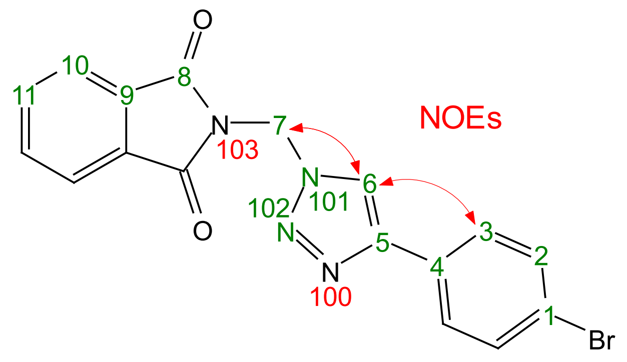

User Report
GOD-GB-168-01

Atom	δ (ppm)	COSY	HSQC	HMBC	NOESY
1 C	122.416			2, 3	
2 C	132.928		2	2	
H	7.590	3	2	1, 2, 4	
3 C	128.356		3	3	
H	7.776	2	3	1, 3, 5	6
4 C	130.861			2	
5 C	147.429			3, 6	
6 C	122.669		6	7	
H	8.292		6	5, 101	3, 7
7 C	51.182		7		
H2	6.158		7	6, 8, 102	6
8 C	167.936			7	
9 C	132.689			10	
10 C	124.630		10	11	
H	7.907	11	10	9, 11	
11 C	135.894		11	10	
H	7.840	10	11	10	
100 N					
101 N	-131.5			6	
102 N	-15.5			7	
103 N					

Remarks:

The sample was prepared by dissolving crystallized material from a broken NMR tube used for crystallization in CD_3CN. The CD_3CN may have contained trace amounts of acetamide due to long-term storage over molecular sieves, although it could also originate from the sample itself.

The NMR data are consistent with the structure shown above. All extracted assignments and correlations are summarized in the table on the right.

Two ^{15}N correlations were observed in the ^1H–^{15}N HMBC spectrum. Based on comparison with the literature (Table 7, Claramunt et al., 1997, *Magn. Reson. Chem.*, 35, 35–75), these signals were assigned to N-101 and N-102. The ^{15}N NMR shifts are reported relative to $MeNO_2$.

P-ID: ML00xxx
Measured on: 01/09/2024
CHIFFRE: GOD-GB-168-01
ELNA#: 13106
Client: Dr. Richard Goddard
Group: XRAY
Spectroscopist: Leutzsch
Analysed on: 03/09/2024
Analysed by: Leutzsch
Amount: 5.0 mg
Solvent: CD3CN
Reference: 1H+13C on solvent,other nuclei w/ xiref
Temperature: 298 K
Spectrometer: av600neo
Probe: cryoBBO
Experiments: 1H-zg30, 13C-zgpg30, 1H-13C-hsqcedetgpsisp2.3, 1H-13C-hmbcetgpl3nd, 1H-1H-cosygpppqf, 1H-1H-noesygpphpp, 1H-15N-hmbcgpndqf

1H{off},1D, 600.20 MHz,CD3CN,298.0K, pulse sequence: zg30

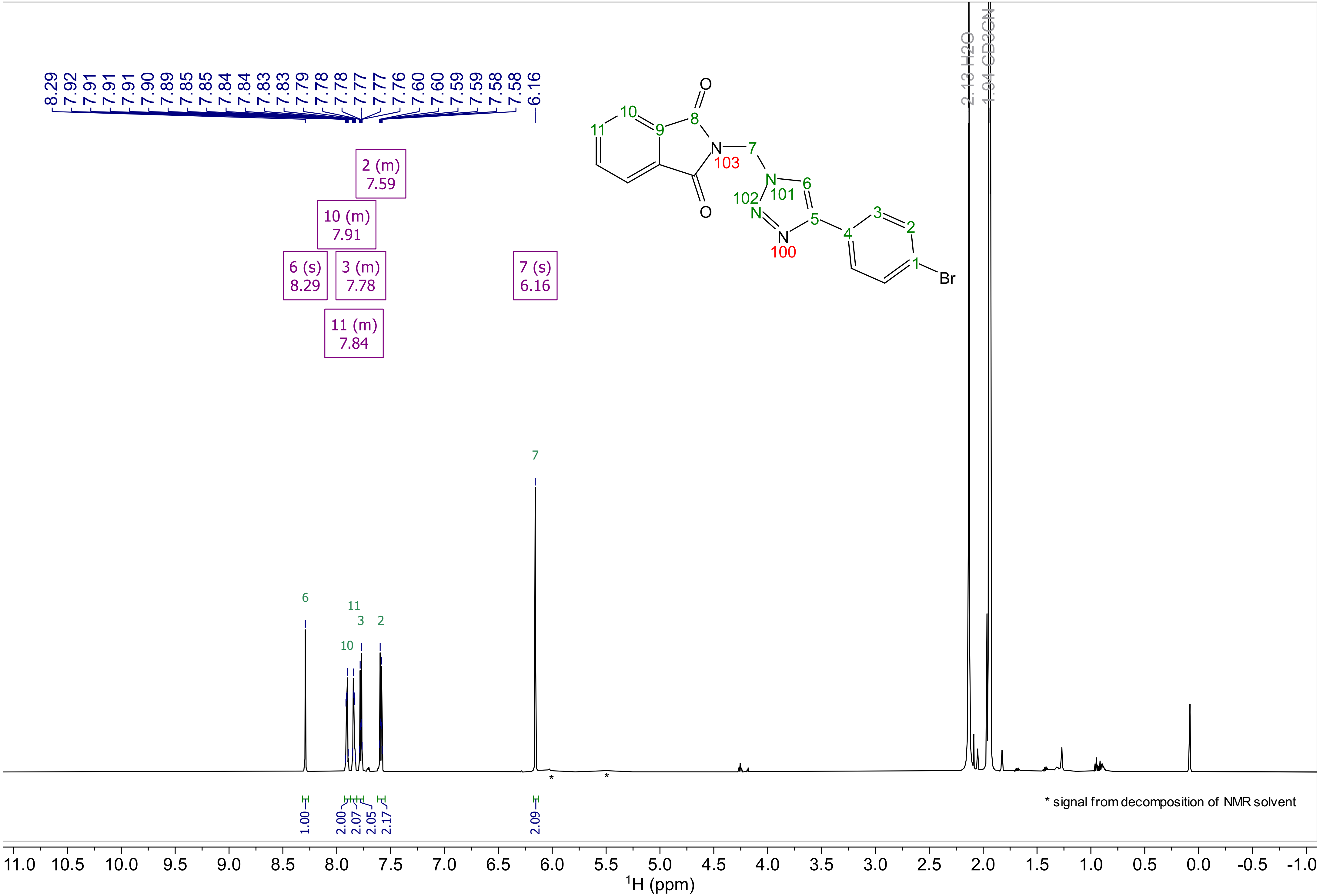

13C{1H},1D, 150.94 MHz,CD3CN,298.0K, pulse sequence: zgpg30

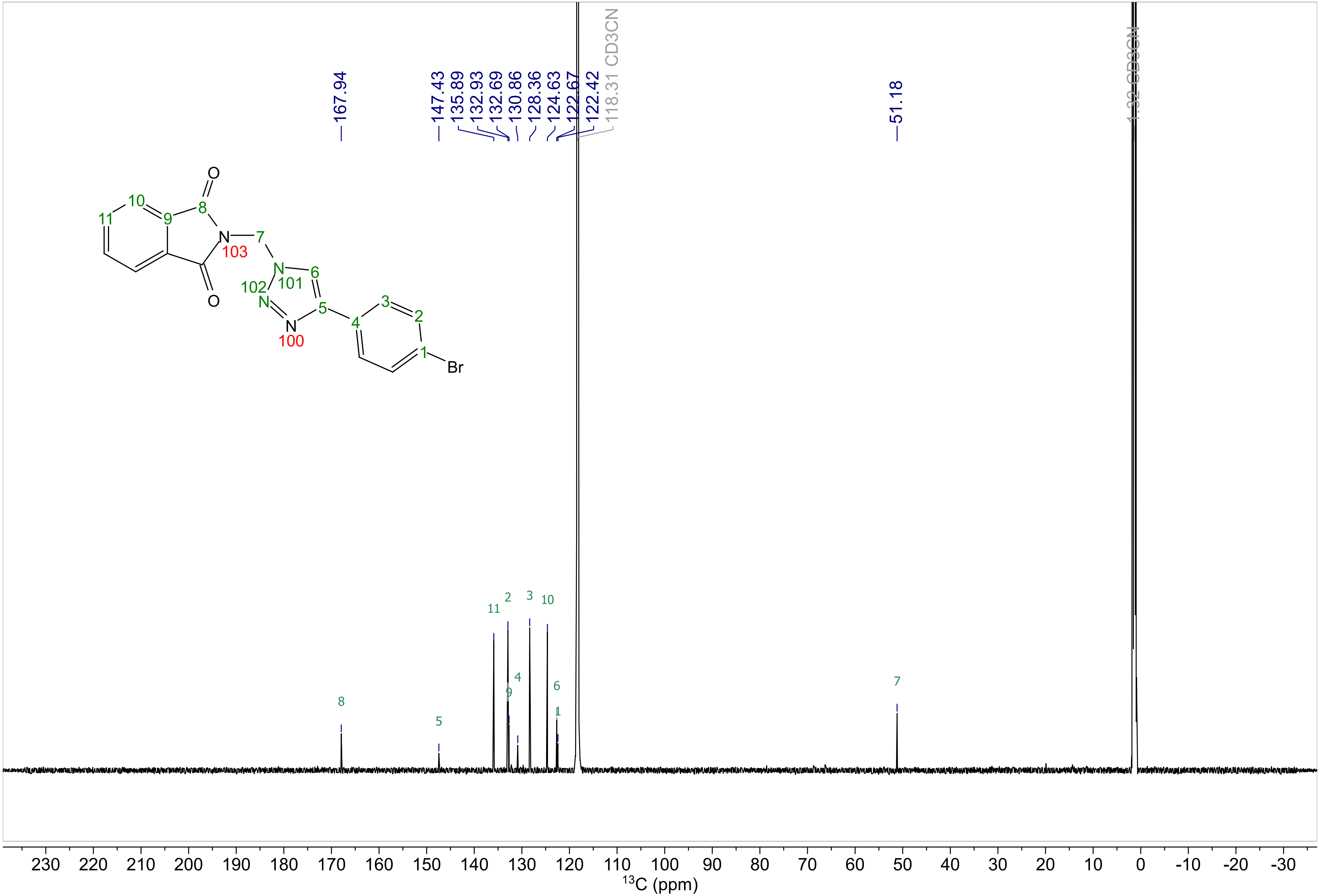

1H{13C},HSQC-EDITED, 600.20 MHz,CD3CN,298.0K, pulse sequence: hsqcedetgpsisp2.3

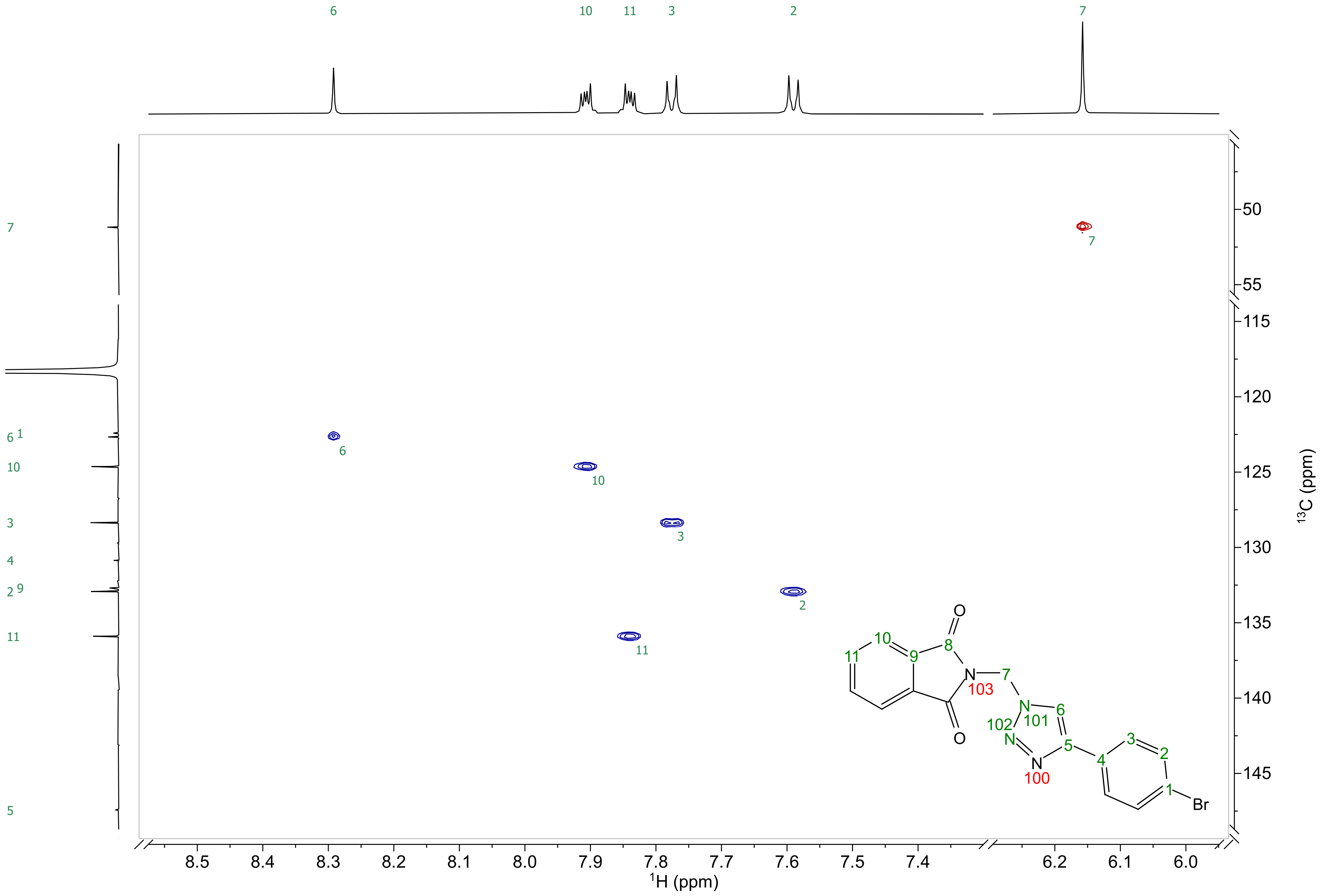

1H{13C},HMBC, 600.20 MHz,CD3CN,298.0K, pulse sequence: hmbcetgpl3nd

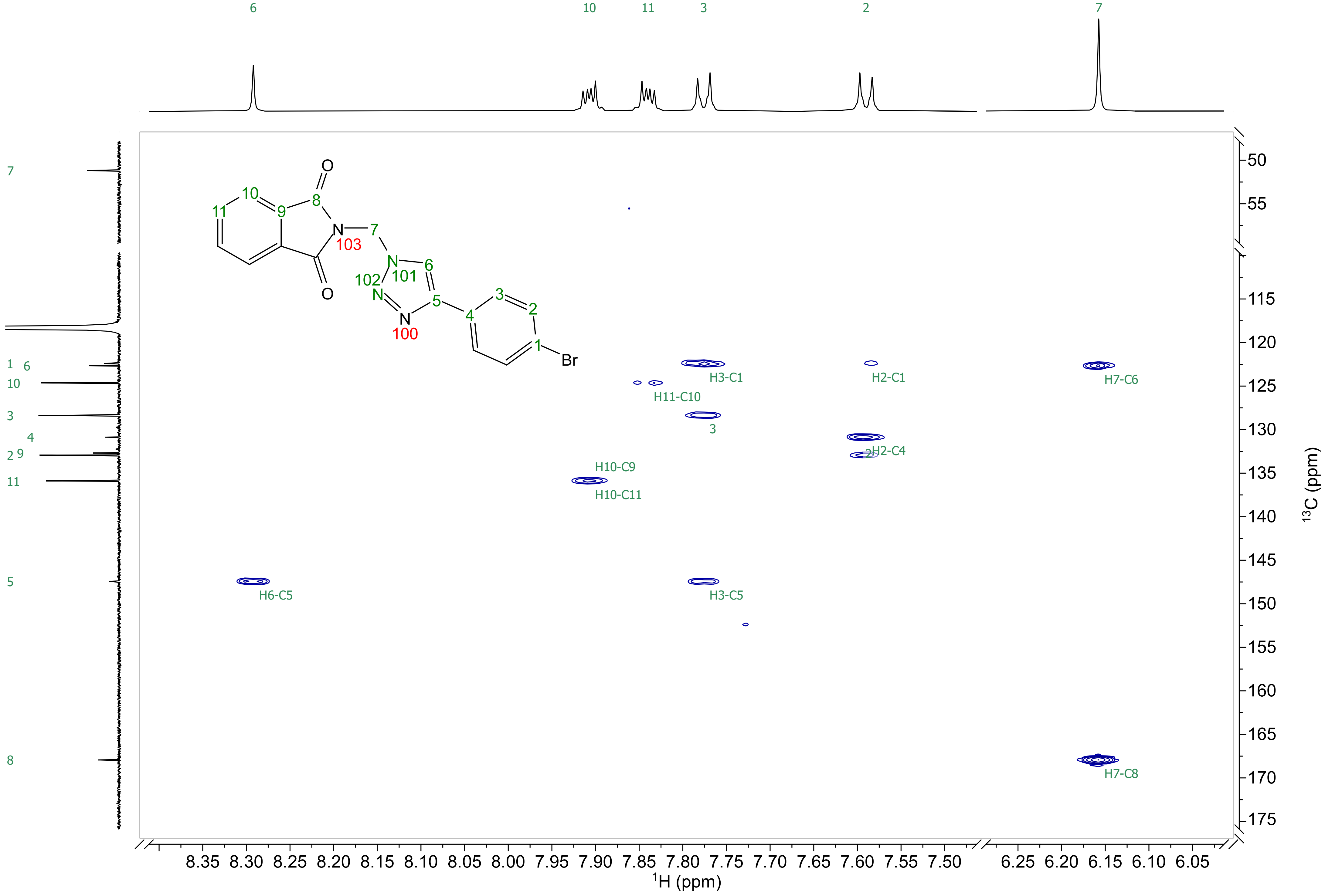

1H{off},COSY, 600.20 MHz,CD3CN,298.0K, pulse sequence: cosygpppqf

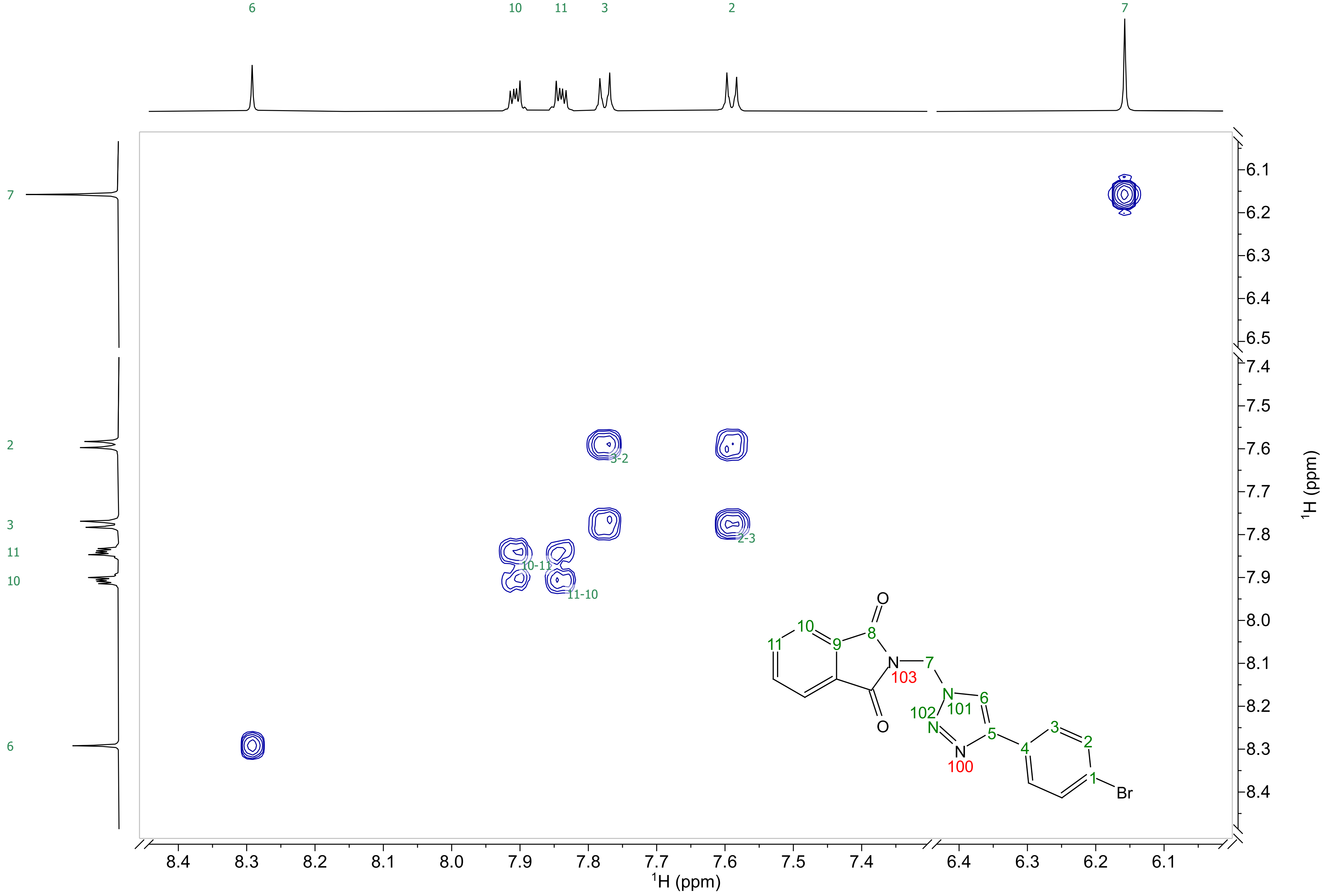

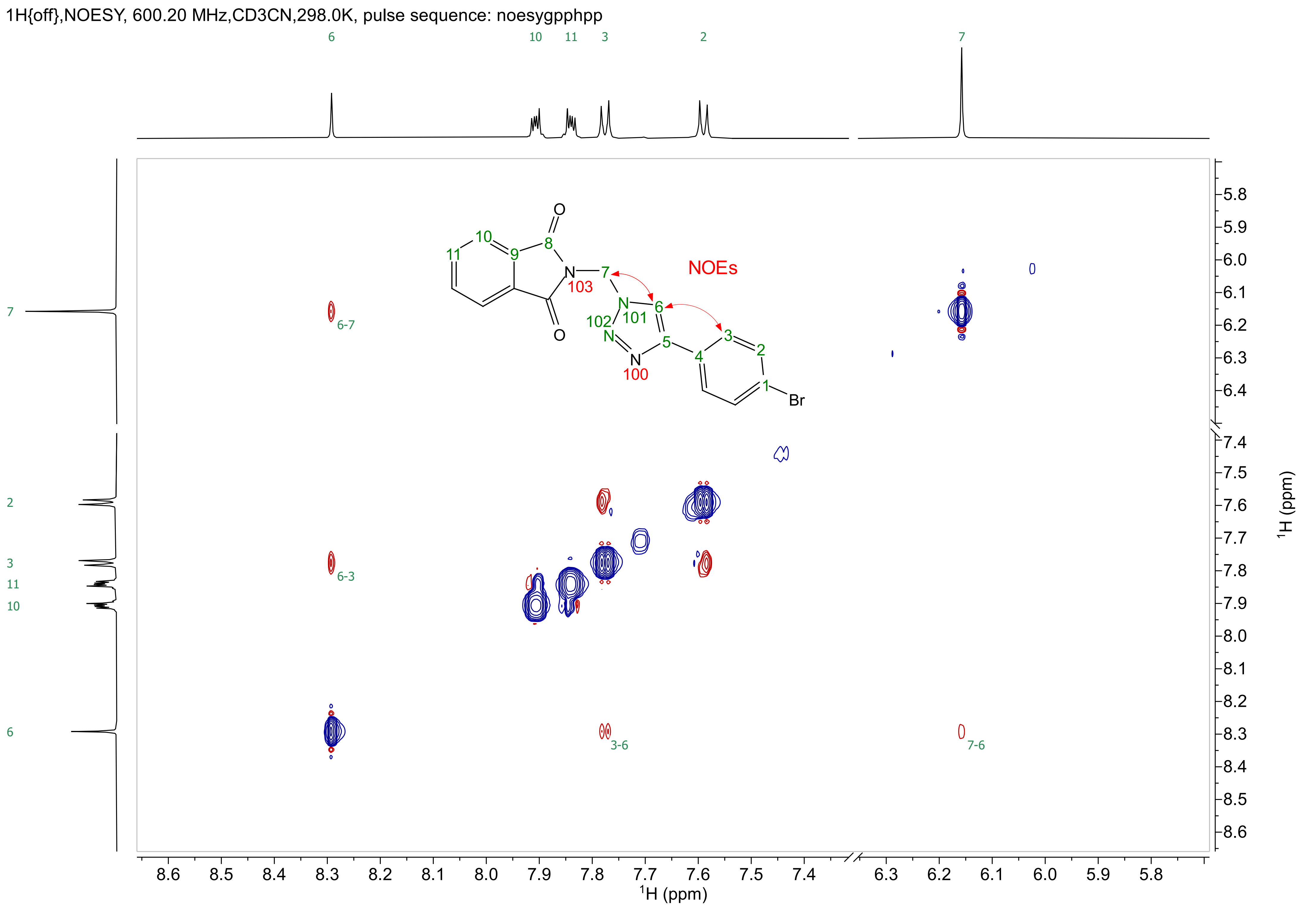
1H{off},NOESY, 600.20 MHz,CD3CN,298.0K, pulse sequence: noesygpphpp
NOEs
6-7
6-3
3-6
7-6
^{1}H (ppm)
^{1}H (ppm)
Br

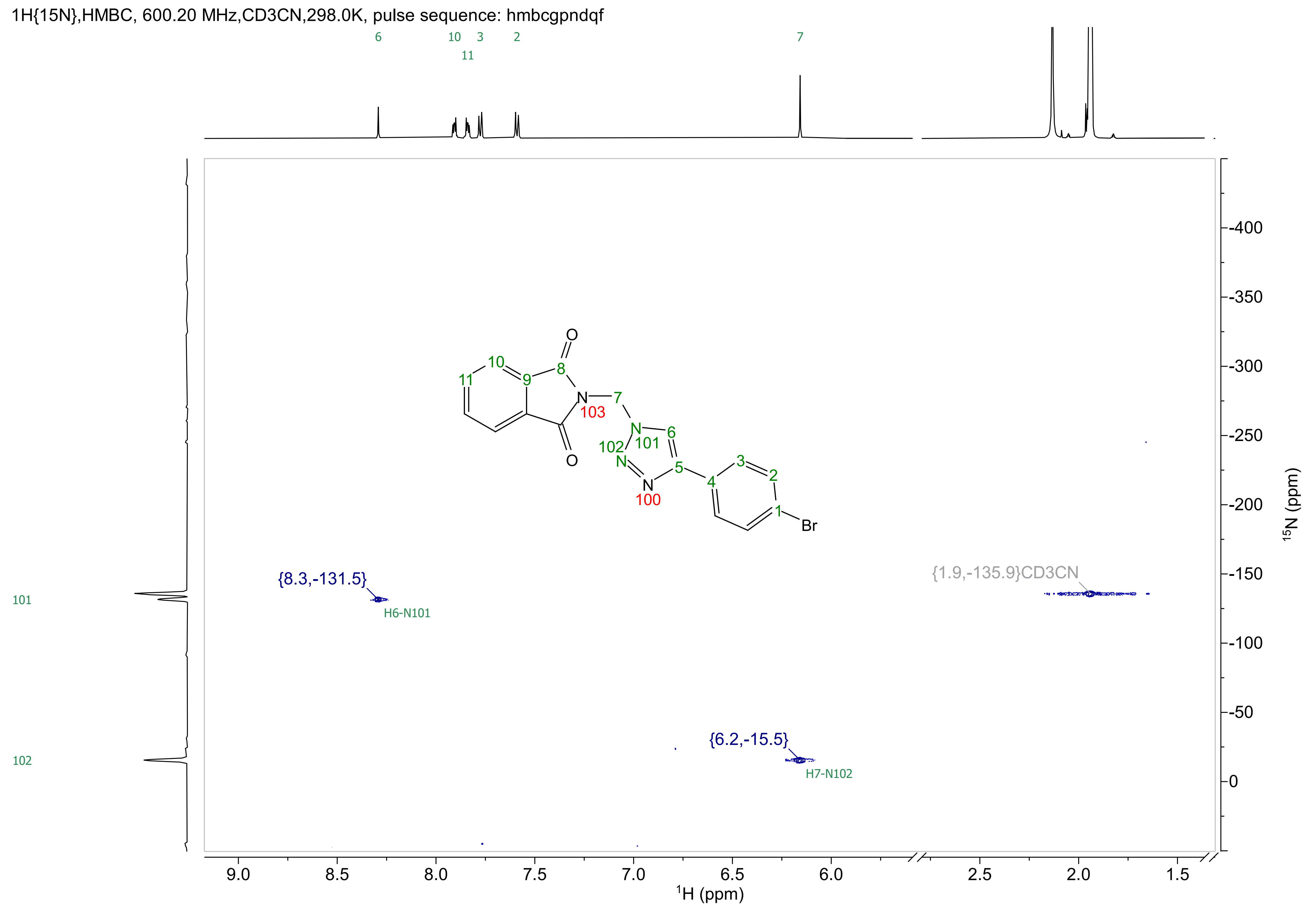
1H{15N},HMBC, 600.20 MHz,CD3CN,298.0K, pulse sequence: hmbcgpndqf
6
10
3
2
7
11
O
10
8
11
9
N
7
103
N
6
102
101
N
5
3
2
O
4
N
100
1
Br
{8.3,-131.5}
H6-N101
{1.9,-135.9}CD3CN
{6.2,-15.5}
H7-N102
101
102
-400
-350
-300
-250
-200
-150
-100
-50
0
15N (ppm)
9.0
8.5
8.0
7.5
7.0
6.5
6.0
2.5
2.0
1.5
1H (ppm)

^{13}C NMR (126 MHz, Chloroform-d) δ 166.68, 147.63, 135.08, 132.14, 131.55, 129.26, 127.53, 124.35, 122.48, 120.74, 49.95.

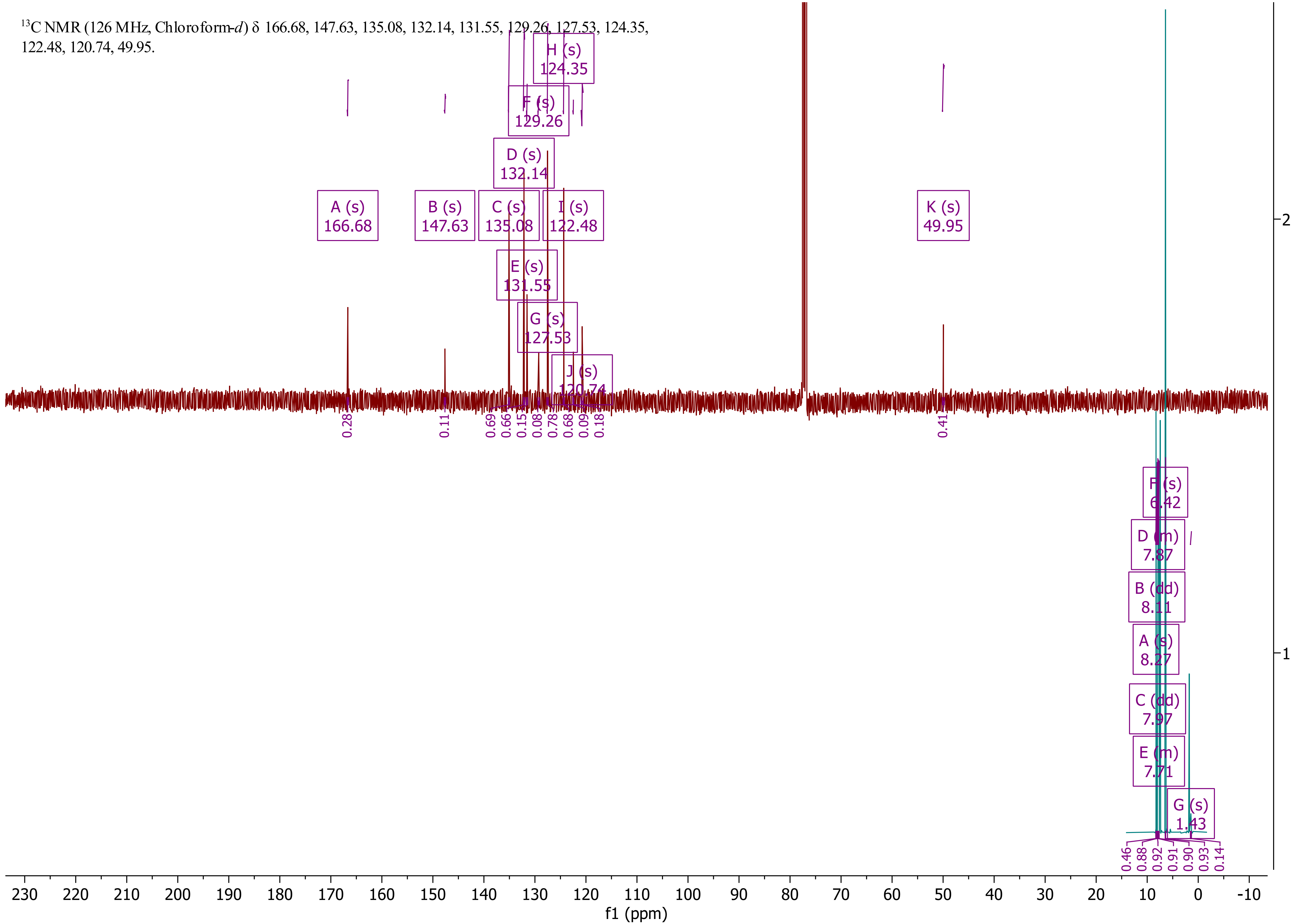

Palme_6064-67_CARBON_2023-11-03_01 — 2023-11-02 — Palme_6064-67 — —

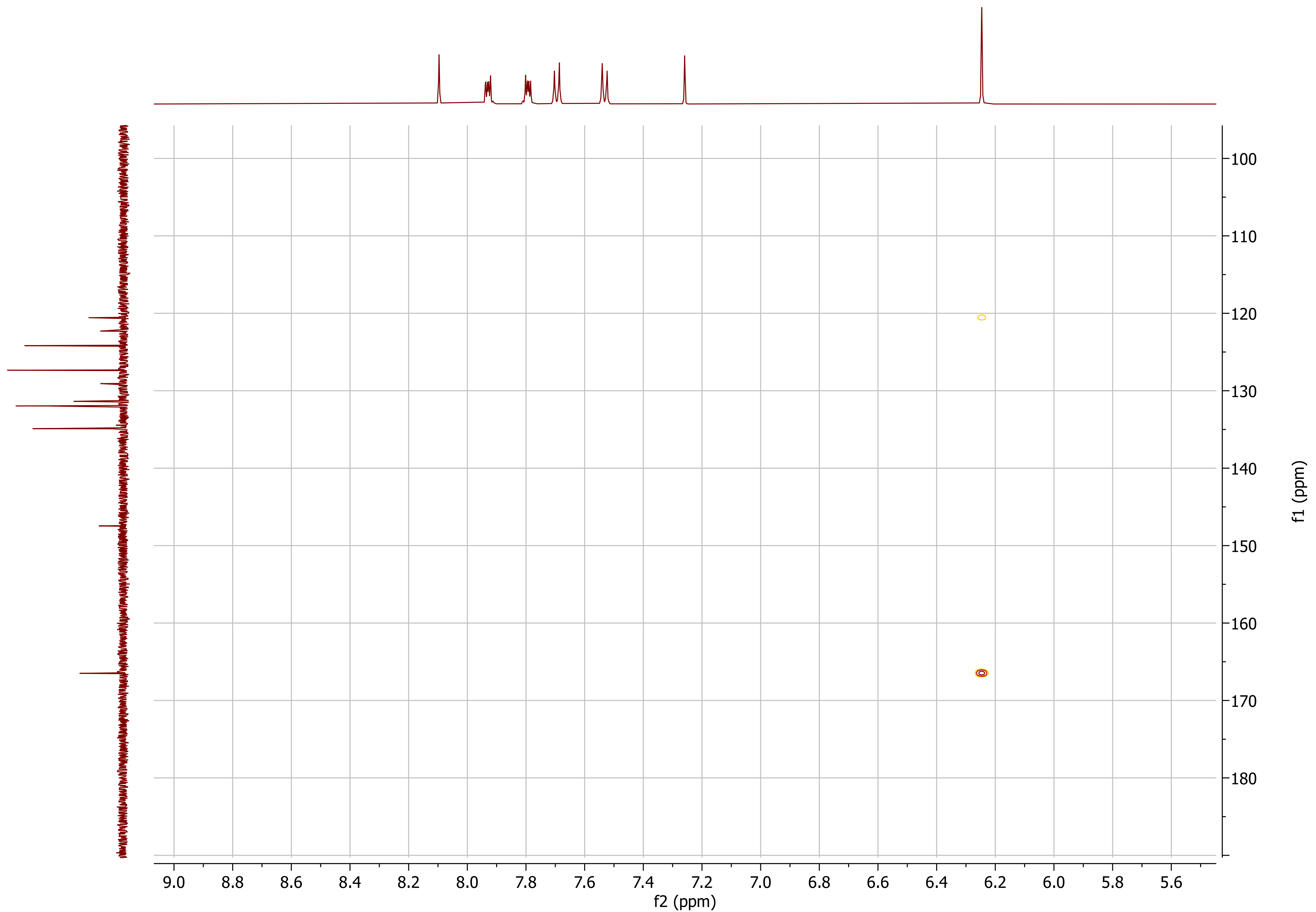

Palme_6064-67_gHMBCAD_2023-11-04_01 — 2023-11-02 — Palme_6064-67 — —

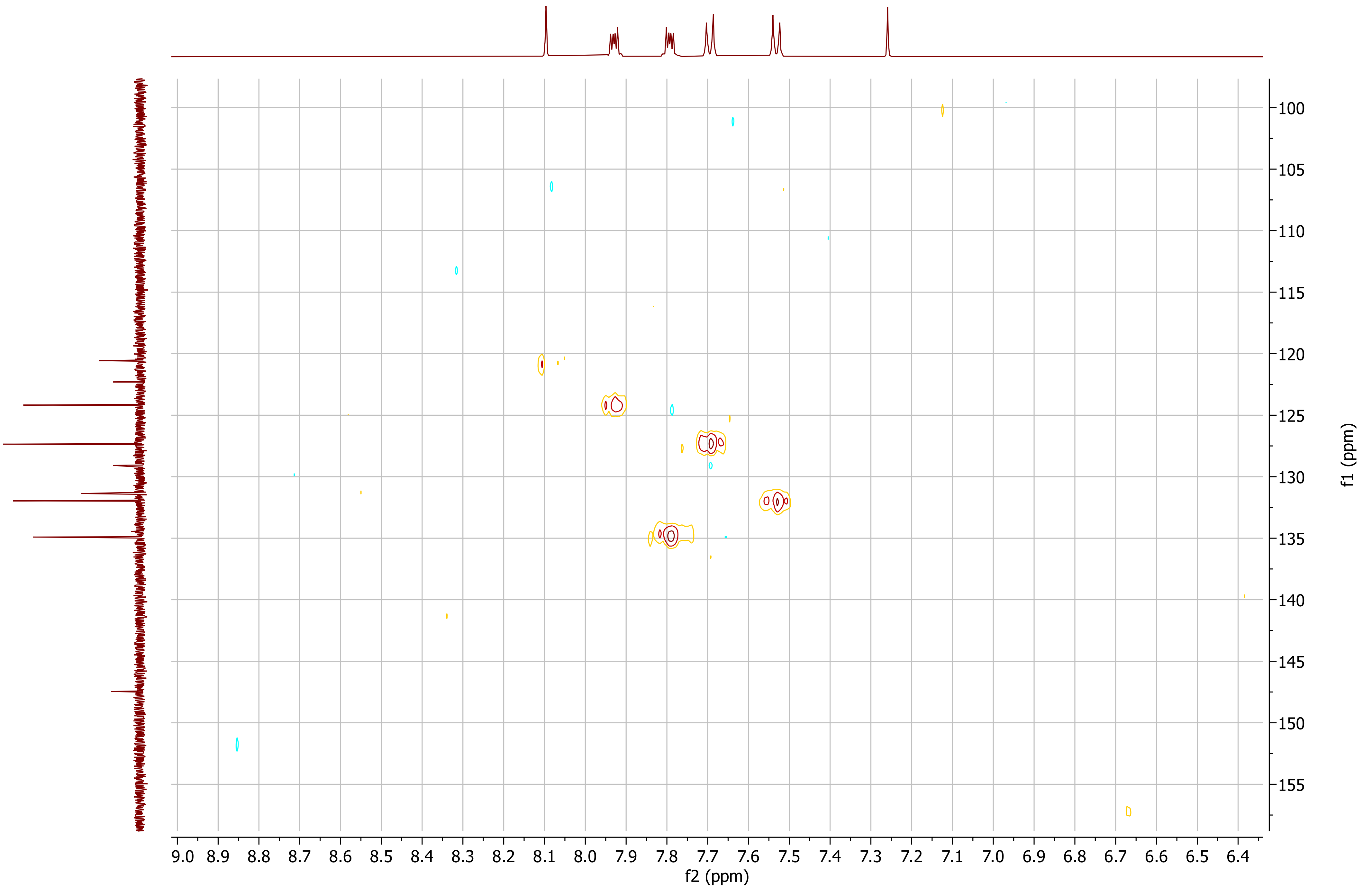

Palme_6064-67_gHSQCAD_PS_2023-11-03_01 — 2023-11-02 — Palme_6064-67 — —

User Report
GOD-GB-173-01

The following 4 components were identified in the sample

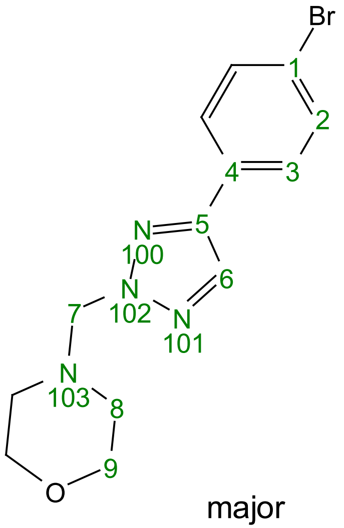

major

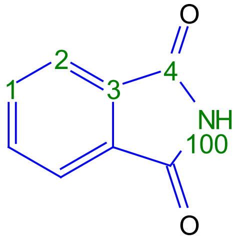

~0.4 eq

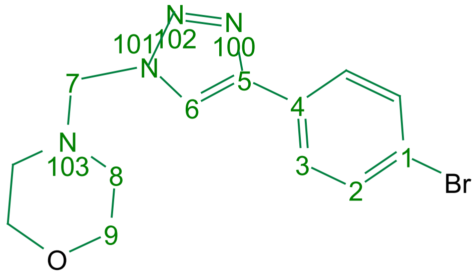

~10 mol%

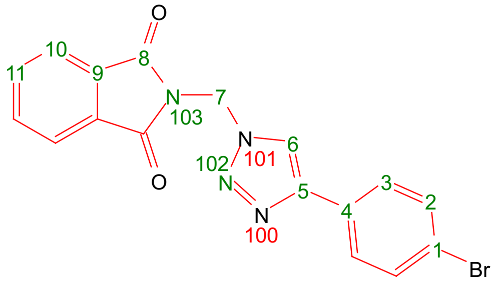

~5 mol%

Remarks:

The NMR sample was prepared by dissolving the remaining crystalline material used for the X-ray analysis in CD_3CN that had been stored over molecular sieves (MS). Unfortunately, not all crystals were fully soluble, so the analysis should not be considered quantitative. It is also possible that the deuterated solvent had partially decomposed, as residual signals of CD_3CONH_2 are visible. However, NMR data obtained for the compounds in chloroform likewise showed broad peaks between 5–6 ppm.

An overview of all chemical shift assignments for the two product isomers is provided on the next page. For the assignment of the starting material, please refer to GOD-GB-168. The NMR data of the major component are consistent with the obtained X-ray structure. A characteristic feature of this compound is the presence of the $^3J(^{15}N)$ $^1H–^{15}N$ HMBC cross-peaks of H-7 with N-100 and N-101. In the other isomer, H-7 shows a ^1H–X HMBC cross-peak to C-6 and N-102. Furthermore, the minor product isomer displays characteristic NOE correlations from H-6 to H-3 and H-7, which are also observed in the starting material. The observed ^{15}N NMR signals of the isomers are in good agreement with reference data reported in the literature (Tables 6 and 7 in Claramunt et al., 1997, *Magn. Reson. Chem.*, **35**, 35–75).

Atom	δ (ppm)	COSY	HSQC	HMBC	NOESY
1 C	122.756			2, 3	
2 C	133.019		2	2	
H	7.625	2	2	1, 2, 4	
3 C	128.626		3	3	
H	7.747		3	1, 3, 5	6, 8
4 C	130.819			2	
5 C	147.434			3, 6	
6 C	132.276		6		
H	8.008		6	5, 101, 102	3, 7, 8
7 C	76.256		7	8	
H2	5.231		7	8, 100, 101, 103	6, 8
8 C	50.914		8	7, 8, 9	
H2	2.612	9	8	7, 8, 9	3, 6, 7, 8, 9
9 C	67.422		9	8, 9	
H2	3.604	8	9	8, 9, 103	8
100 N	-56.4			7	
101 N	-45.1			6, 7	
102 N	-120.3			6	
103 N	-331.2			7, 9	

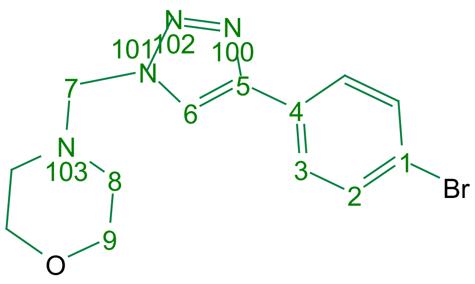

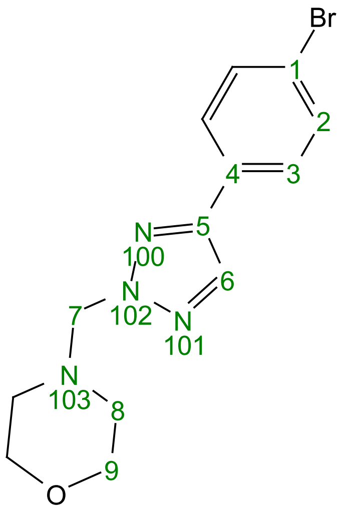

Atom	δ (ppm)	COSY	HSQC	HMBC	NOESY
1 C	122.144			2, 3	
2 C	132.913		2	2	
H	7.614	2	2	1, 2, 4	
3 C	128.288		3	3	
H	7.793		3	1, 3, 5	6
4 C	131.361			2	
5 C	146.703			3	
6 C	122.604			7	
H	8.135		6	5, 101, 102	3, 7, 8
7 C	72.087		7		
H2	5.210		7	6, 8, 100, 103	6, 8
8 C	50.839		8	7, 8, 9	
H2	2.562	9	8	8, 9	6, 7, 9
9 C	67.319		9	8, 9	
H2	3.622	8	9	8, 9, 103	8
100 N				7	
101 N					
102 N	-14.6				
103 N	-333.2			7, 9	

P-ID: ML00xxx
Measured on: 01/09/2024
CHIFFRE: GOD-GB-173-01
ELNA#: 13107
Client: Dr. Richard Goddard
Group: XRAY
Spectroscopist: Leutzsch
Analysed on: 04/09/2024
Analysed by: Leutzsch
Amount: 15.0 mg
Solvent: CD3CN
Reference: 1H+13C on solvent,other nuclei w/ xiref
Temperature: 298 K
Spectrometer: av600neo
Probe: cryoBBO
Experiments: 1H-zg30, 13C-zgpg30, 1H-13C-hsqcedetgpsisp2.3, 1H-13C-hmbcetgpl3nd, 1H-1H-cosygpppqf, 1H-1H-noesygpphpp, 1H-15N-hmbcgpndqf

1H{off},1D, 600.20 MHz,CD3CN,298.0K, pulse sequence: zg30

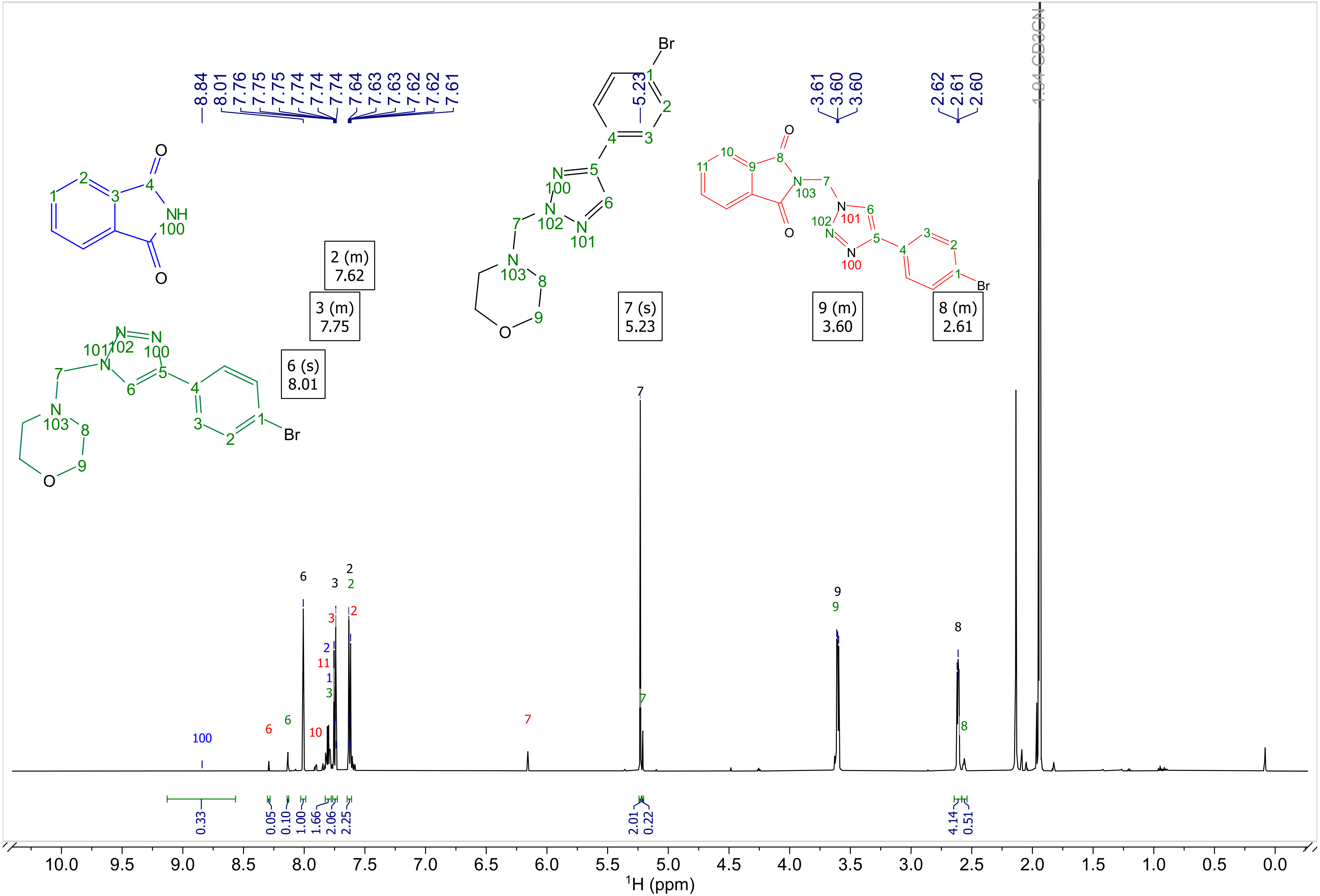

1H{off},1D, 600.20 MHz,CD3CN,298.0K, pulse sequence: zg30

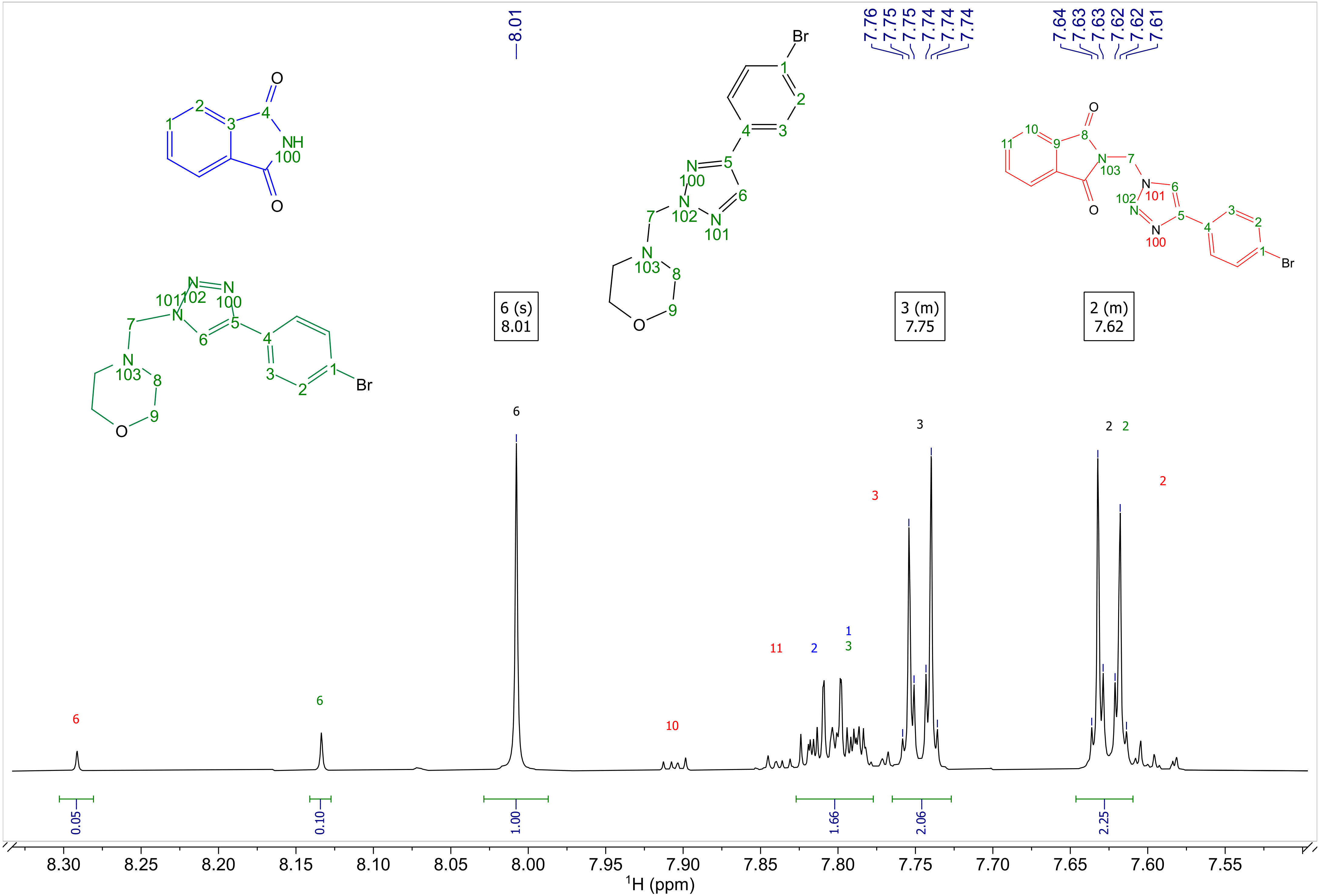

13C{1H},1D, 150.94 MHz,CD3CN,298.0K, pulse sequence: zgpg30

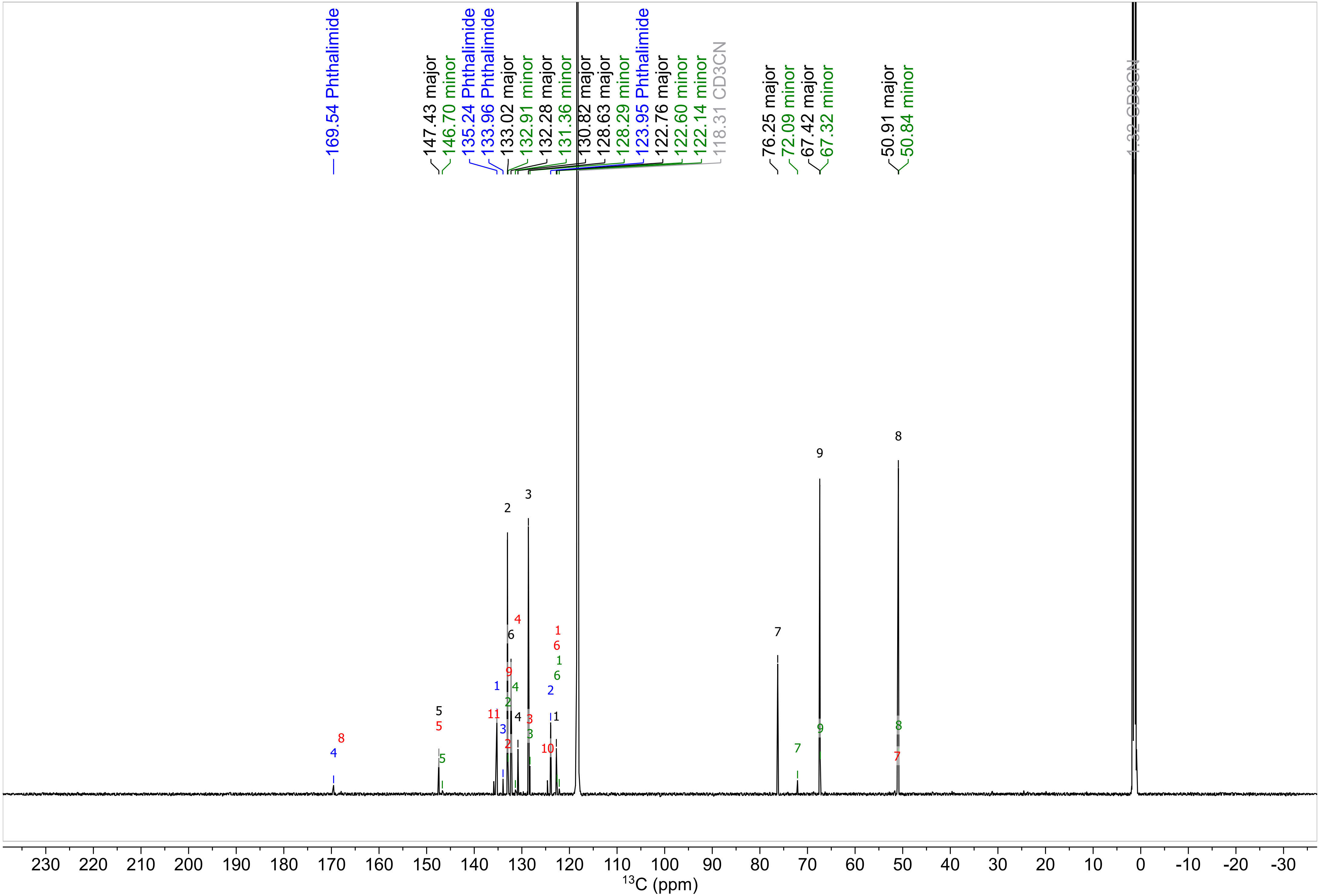

1H{13C},HSQC-EDITED, 600.20 MHz,CD3CN,298.0K, pulse sequence: hsqcedetgpsisp2.3

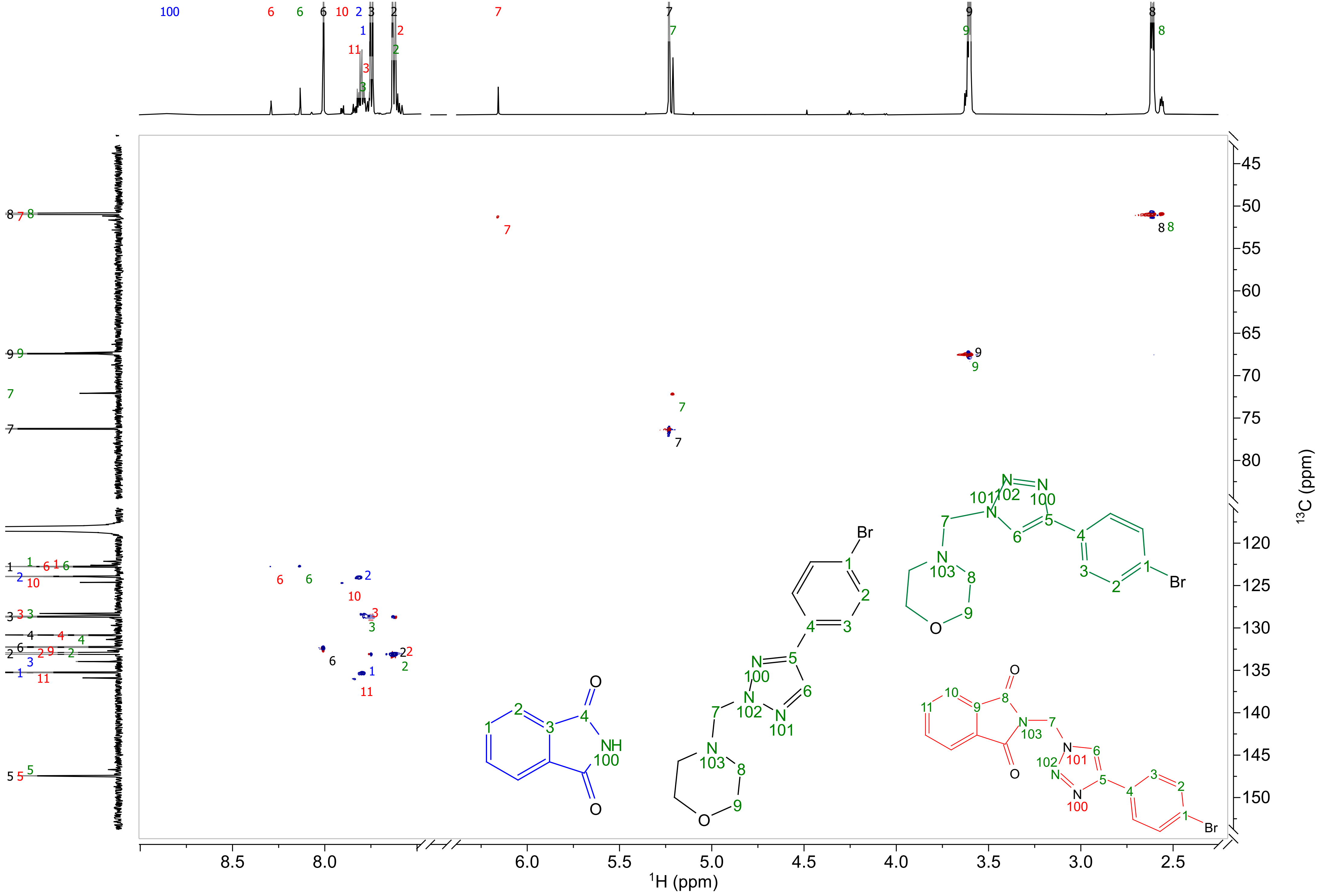

1H{13C},HMBC, 600.20 MHz,CD3CN,298.0K, pulse sequence: hmbcetgpl3nd

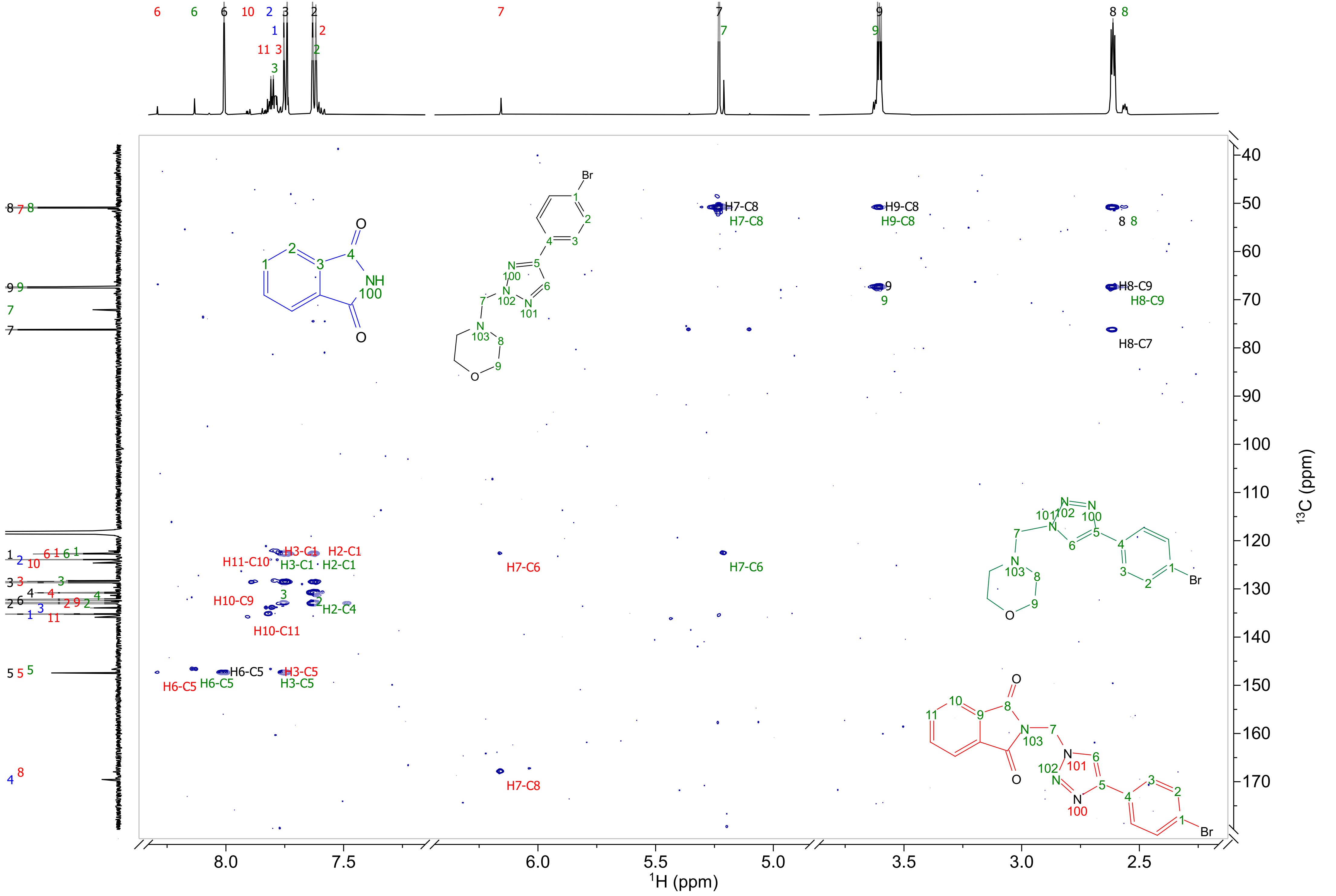

1H{off},COSY, 600.20 MHz,CD3CN,298.0K, pulse sequence: cosygpppqf

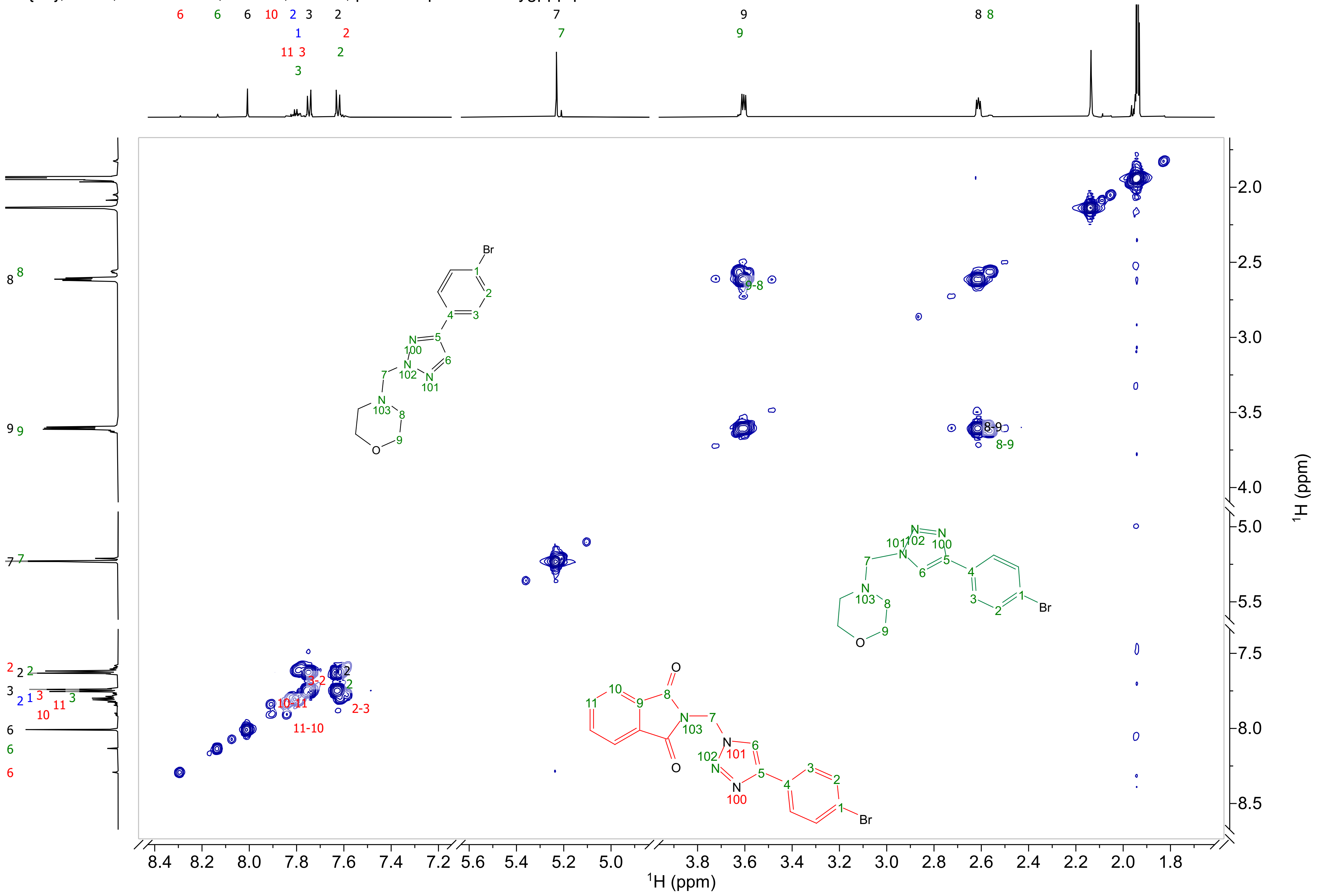

1H{off},NOESY, 600.20 MHz,CD3CN,298.0K, pulse sequence: noesygpphpp

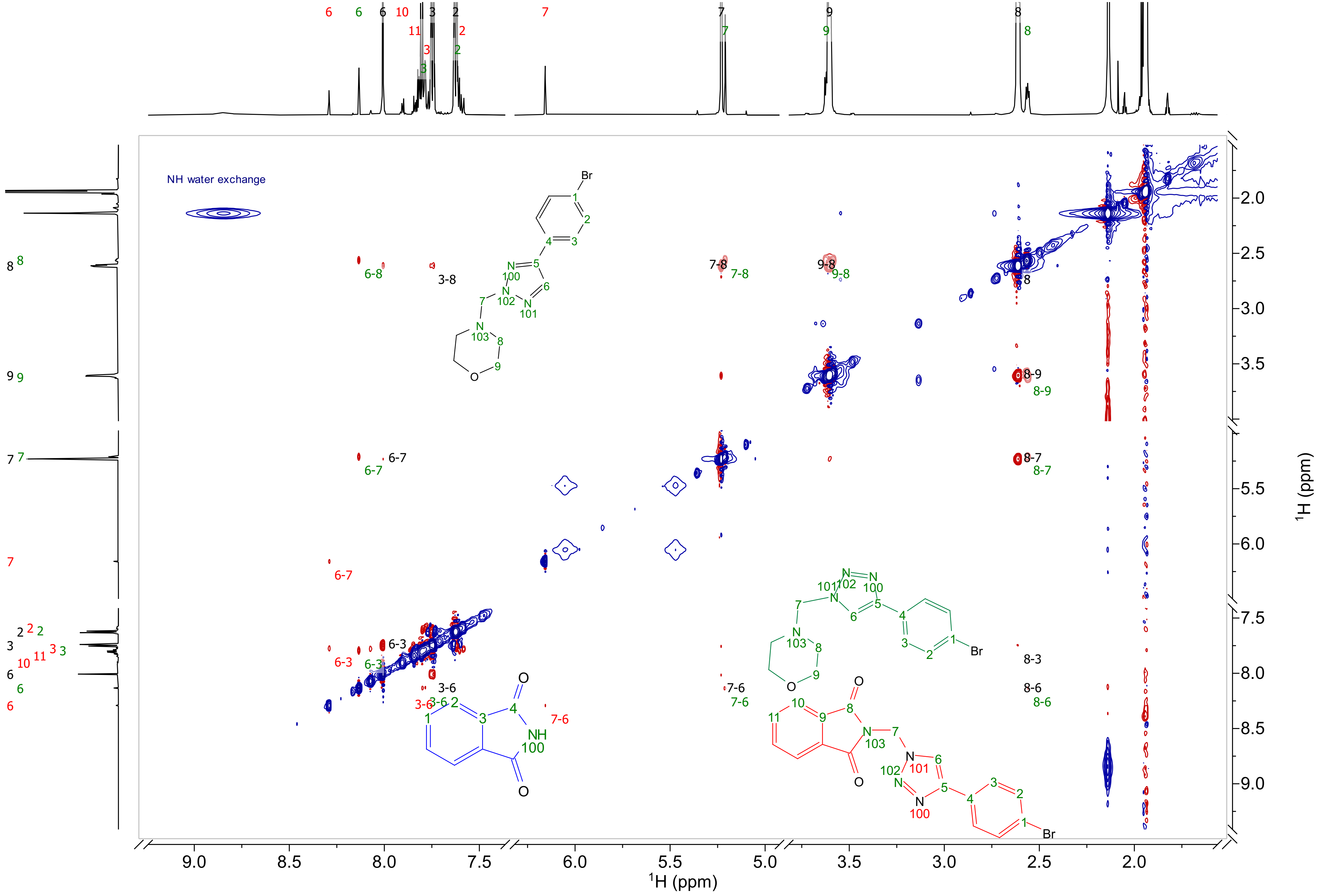

1H{15N},HMBC, 600.20 MHz,CD3CN,298.0K, pulse sequence: hmbcgpndqf

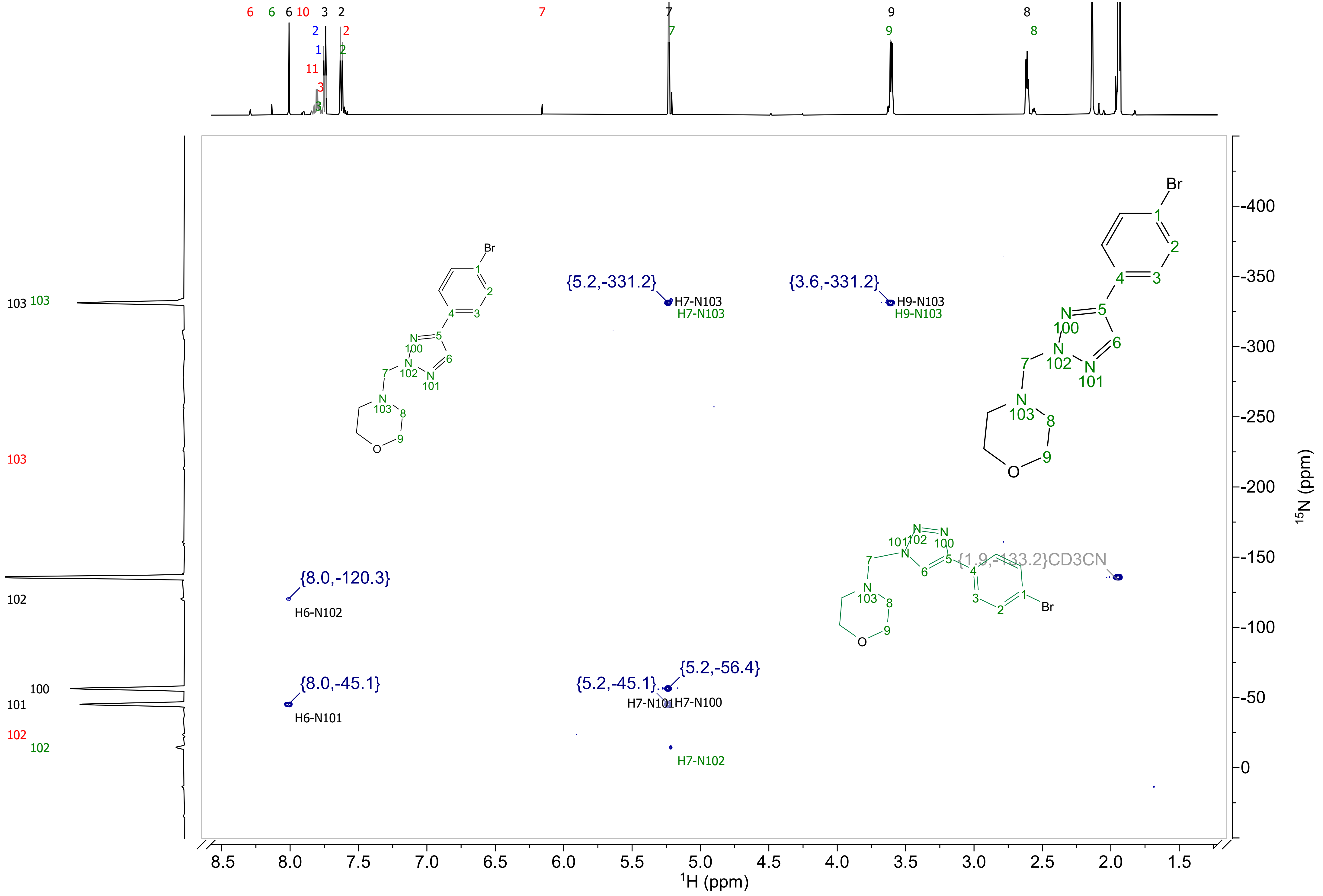

Supplement: Supplementary file 9 [file c-82-00144-sup9.pdf]
